# A Memoir on the Life and Character of Philip Syng Physick, M. D.

**Published:** 1839-04-27

**Authors:** J. Randolph


### MEDICAL EXAMINER.

DEVOTED TO MEDICINE, SURGERY, AND THE COLLATERAL SCIENCES.

No. 17.] PHILADELPHIA, SATURDAY, APRIL 27, 1839. [Vol. II.

### A MEMOIR ON THE LIFE AND CHARACTER OF PHILIP SYNG PHYSICK, M. D.,

By J. Randolph, M.D.

[Read before the Philadelphia Medical Society, Feb. 20, 1839.]

Gentlemen,—Permit me to express my sincere acknowledgments for the honour you have conferred, in appointing me to prepare a memoir of the life and character of the long venerated President of this institution, the late Doctor Physick.

I am quite sensible, that the selection was owing rather to my connexion with the illustrious deceased, and the close and intimate relation which necessarily existed between us for a long series of years, than to any peculiar ability I may possess, of recording his many virtues and high qualifications. I am fully aware also, of the weighty responsibility which that man assumes, who undertakes to transmit to posterity a portrait, which, well and properly executed, may serve as a light and example to illumine and instruct succeeding ages. The effort to accomplish this object I consider, however, a duty which I owe alike to you, and to the memory of Dr. Physick; and I shall endeavour to discharge my obligations in the best possible manner consistent with my means and abilities.

Most deeply do I deplore, in the commencement of my task, the want of proper materials, which, faithfully recorded, would enable Dr. Physick's great and exalted character fully to develope itself. Many of you, gentlemen, cannot be ignorant, that the subject of our memoir throughout his whole life, entertained a most invincible repugnance to appear before the public in the shape of an author. Sorry I am to say, that this feeling existed with him to the day of his death, and induced him to make an ardent request, and exact the promise that none of his manuscript lectures or letters should be made public.

The same modesty of feeling which he possessed to an extraordinary degree, and which forms so principal an ingredient in the composition of a truly great and noble mind, caused him also to refuse to comply with the repeated requests which were made to him, to furnish sufficient facts upon which a sketch of his biography might be founded. Upon one occasion only, after urgent solicitations on my part, did he place in my possession some dates and incidents of his life, with the permission that I might make use of them; he excused himself, however, from completing the materials at that time, upon the plea of his ill health, and a promise to furnish them at a subsequent period. His disinclination to fulfil this promise was so obvious, that I did not feel myself justifiable in renewing the application.

Philip Syng Physick was born in Philadelphia on the 7th of July, 1768. His father, Mr. Edmund Physick, was an Englishman, and was characterized for possessing strong mental powers, with which were united strict integrity of principle, and considerable knowledge of the world. Previously to the separation of the United States from Great Britain, he held the office of Keeper of the Great Seal of the Colony of Pennsylvania; and subsequently to the Revolution he took charge of the estates belonging to the Penn family, and served as their confidential agent. Doctor Physick's mother was a most estimable, pious woman, who was blessed with a strong intellect, and evinced throughout her life, great judgment and decision of character. The Doctor never ceased to feel and express, as long as he lived, the greatest filial love and reverence for these honoured parents. We have frequently known him to declare, that he was convinced that whatever was most useful and excellent in his character, was attributable to the early lessons and impressions which he imbibed from them.

From such parents as these it must have resulted that the greatest care and attention would be bestowed upon the education of their children. It must be regarded as a fortunate circumstance, also, that his father had succeeded by great industry and attention to business, in accumulating a property which, in those days, was looked upon as considerable; and being thus in possession of ample means, he was enabled to carry out to the fullest extent, the plan of education which he designed for his son.

In doing so, Dr. Physick informed me that his father was influenced by a degree of liberality very unusual in that, or indeed in any age. Double fees transmitted to the teacher uniformly testified the great importance which he attached to a liberal education, and the value which he thought should be set upon the sources from which it emanated. This was not only intended for an encouragement to the instructer to use his best endeavours on behalf of his son, but because the donor believed it to be his duty to increase the remuneration, inasmuch as the charges for tuition in that day were so low that they could not be considered as a fair equivalent for the services rendered.

Mr. Physick placed his son, when eleven years of age, in the academy belonging to the Society of Friends, in South Fourth street, under the tuition of Robert Proud. At this period Mr. Physick resided in the country, upon the banks of the Schuylkill, several miles from the city, upon an estate belonging to the Penn family. In order to facilitate the education of his son, he was boarded in the city, in the family of the late Mr. John Todd, the father-in-law of the present venerable Mrs. Madison. Even at that early age, the subject of our memoir exhibited very strong indications of those well-regulated habits of order and method which adhered to him so closely throughout his life. In consequence of his family residing in the country, he was permitted to go home every Saturday after the school broke up, for the purpose of visiting them and remaining with them until the following Monday morning. He then not unfrequently was obliged to walk into town, and sometimes through most inclement weather. Notwithstanding this, he always succeeded in presenting himself at school exactly at the time of its opening. His teacher was so much gratified with this extraordinary punctuality, that he took pleasure in holding him up as an example to other boys, who, though living in the vicinity of the school, were too apt to be remiss in making their appearance at the proper hour.

Young Mr. Physick remained at this academy until he entered the collegiate department of the University of Pennsylvania. He then passed through the usual course of studies prescribed in that institution, and took the degree of Bachelor of Arts in May, 1785. I am not aware that any thing remarkable occurred during the period of his collegiate studies. That he was a diligent and exemplary student, cannot for a moment be questioned. It is well known that he was particularly successful in acquiring a thorough and intimate knowledge of the classics, of which he retained sufficient, amid all his engagements, to be able to translate them with facility, to the time of his death.

In June, 1785, one month after he obtained the degree of Bachelor of Arts, he commenced the study of medicine, under the superintendence of the late Doctor Adam Kuhn, well known as the pupil of Linnæus, and a most distinguished and successful practitioner, and then Professor of the Theory and Practice of Medicine in the University of Pennsylvania. Of the particular motives which influenced young Mr. Physick in the choice of this profession, I am unable to speak. It does not appear that he at that period evinced any strong predilection for this department of science. I think it more than probable that he was principally governed by the wishes of his father; and so strong were his feelings of filial obedience, that I am very certain that he would at any time readily have yielded his own wishes to those of his parents. The following anecdote is traditionary in the family. His father, whilst handling a knife, had the misfortune to cut one of his fingers; and the wound proved to be so severe that he was obliged to engage the services of a medical friend. Upon one occasion his son begged of him to be permitted to apply the necessary dressings and bandage to the finger: his father consented, and was so much surprised at the great skill and dexterity which his son displayed in making the applications, that he determined in his own mind to make him a surgeon.

If it be true that we are indebted so exclusively to Mr. Physick for directing his son's attention to the study of medicine, to what an immeasurable extent does it not increase the amount of obligation and gratitude that we owe to him?

Dr. Physick was remarkable throughout life for possessing feelings of the most acute and susceptible nature. It may be truly said of him that he possessed a soul feelingly alive to the miseries and sufferings of others. I feel compelled to confess, that I do not think Dr. Physick himself could support pain with the same degree of fortitude and composure which we have sometimes met with in persons who suffered to an equal extent with himself; it is undeniable, however, that he was extremely unwilling to be the source of inflicting pain upon others. This tenderness of feeling, which adhered to him closely as long as he lived, as I shall have occasion to show during the progress of this memoir, existed also in full force in the days of his youth. He used frequently to declare, at this period of his life, that he never could be a surgeon. Little was he aware, that he would live to afford in his own person, a complete illustration of the position, that the practice of medicine and surgery, so far from hardening and rendering callous the feelings, has a direct contrary tendency, and serves, pre-eminently, to soften and refine them. His example also went far to prove, in connection with the result of our whole experience upon this subject, that in order for a man to become a great and good surgeon, it is absolutely necessary for him to possess, to the fullest extent, the best and kindest feelings of which human nature is susceptible.

The following incident, which occured to Dr. Physick, and which was in fact characteristic, may not be deemed uninteresting. Soon after he commenced the study of medicine, it was announced that an amputation would be performed upon a certain day, at the Pennsylvania Hospital. His preceptor, Professor Kuhn, wished him to witness this operation, but understanding perfectly well the peculiar temperament of his pupil, he gave it as his advice that his father should accompany him. His father did go with him, and fortunately too, inasmuch as his son became so sick during the operation, that it was necessary to lead him from the amphitheatre before it was concluded.

Dr. Physick continued to prosecute his medical studies under the superintendence of Professor Kuhn, for the period of three years. In those days it was customary for the student of medicine, previously to his obtaining the honours of the doctorate, to go through a much more extensive course of reading than is now deemed necessary. By the direction of his preceptor, Dr. Physick read through, most diligently and faithfully, many voluminous works of the older medical writers, some of which, if not absolutely obsolete at the preseut day, are only used as works of reference. We have abundance of evidence which goes to prove, that even at that early period of his life, Dr. Physick evinced the most resolute determination to qualify himself by every possible means, for assuming a most useful and honourable standing in his profession: and there cannot be a question but that he must have gleaned from amidst this great mass of laborious reading, much valuable information, which he subsequently applied to a most excellent purpose.

It may be stated that Dr. Physick's whole deportment during the period of his pupilage with Professor Kuhn, was so perfectly correct and satisfactory, as to merit his entire approbation: it is well known, too, that Dr. Physick cherished, as long as he lived, feelings of the warmest affection and regard for his venerable preceptor, and it was a source of great gratification to him to know that these feelings were reciprocated.

In addition to the instruction which Dr. Physick derived from Professor Kuhn, he also attended at this same period, the medical lectures delivered in the University of Pennsylvania. He did not, however, graduate in medicine in that institution. The opportunities for the acquisition of profound medical knowledge offered by the schools and hospitals of this country, then in its infancy, were too limited to satisfy either his conscience or his ambition. He could not convince his mind that his knowledge of medicine was sufficiently enlarged to warrant him in assuming to himself the deep and important responsibilities attendant upon the practice of a profession which involved the lives and happiness of so many of his fellow creatures. In order for the more effectual completion of his education, he entertained an ardent desire to visit Great Britain, and avail himself of the advantages which were afforded by the great schools and hospitals of London and Edinburgh. His father happily coincided with these views, and determined upon accompanying his son. Accordingly, they embarked for Europe in November, 1788, and arrived in London in January, 1789.

I may mention that Dr. Physick's sole object in visiting Europe, was that of acquiring medical information. I doubt very much whether any man ever visited that country with less desire or expectation of partaking of its gaities and amusements than himself. I repeat, with him the grand consideration was the acquisition of knowledge: to this he applied himself with the most ardent devotion, and never permitted amusements of any kind to turn him aside from the pursuit of it.

Fortunately for Dr. Physick, his father's connections in London were such, that he was enabled to introduce his son to some of the most learned and polished society, both among the nobility and gentry, of that great metropolis. An intercourse of this kind created for him an influence, and gave him opportunities by means of which his cherished views were considerably promoted. Any one who ever encountered Dr. Physick, must have been struck with the exceeding dignity and courteousness of his manner. For this no doubt he was principally indebted to nature. I am, however, of the opinion that it was in some degree acquired and confirmed by his association with the most elevated society whilst abroad. By means of this same influence, Mr. Physick succeeded in securing the consent of Mr. John Hunter, then one of the most celebrated anatomists and surgeons of the age, to receive his son under his immediate care and tuition.

I have reason to believe that Dr. Physick considered this as the most important era in his professional life. He early became convinced of the extraordinary advantages which he might derive from this connection with Mr. Hunter, and proceeded accordingly to devote himself with the most ardent zeal to the study of practical anatomy and surgery. By dint of constant and unwearied application to his studies, aided also by a course of unceasing and untiring dissections, he soon made rapid advancement in the attainment of his objects, and what was also of much consequence, secured to himself the approbation and esteem of his great master. Mr. Hunter, in fact, was so well pleased with the zeal and industry, combined with the correct deportment, exhibited by Dr. Physick, that he took pleasure in acknowledging him as a favourite pupil, and bestowed upon him, in the most unreserved confidence, the full benefit of his advice and experience. During this period, Dr. Physick attended regularly the lectures delivered by Mr. John Clark, and Dr. William Osborne, on Midwifery.

Among the manuscript papers left by Dr. Physick, which have fallen into my possession, I have a note book, kept by him during his stay in England, in which he recorded such facts and incidents as came under his observation, which he supposed might be of service to him subsequently. I take the liberty of making two or three extracts from these notes, in order to exemplify the careful manner in which he performed this duty, and the pains which he took to treasure up all the information which he gained.

"February, 1789.—Visited Mr. Hunter. In the evening, after being entertained with tea, coffee, and general conversation, Doctor Baillie exhibited a preparation." He then goes on to describe the preparation; which, although exceedingly interesting to the medical profession, it would not be proper to insert here.

"February, 1789.—Mr. Home performed an operation on a sheep which had the staggers, in the following manner. After making a crucial incision through the integuments of the cranium, he applied the trephine, and removed a portion of the bone from the upper and middle part of the cranium. When this was done, he introduced a pair of small forceps, with which he extracted a tænia hydatigena. The effect was, that the sheep, being set at liberty, stood on its legs, which before it could not do. This, however, was only a temporary amendment, as it died about twenty hours after the operation was performed."

"November 15, 1789.—Mr. Cruickshank related the particulars of a case of hydrothorax, in which, upon opening into the right side of the chest, he evacuated nine pints of water, and in the left side there was found one pint. The lung of the right side was compressed to a small size, and instead of feeling spongy, as common, it was solid and fleshy, and quite incapable of being dilated by air, so that the respiration was carried on by the left lung altogether. The patient, during his life, was incapable of sitting or standing up, feeling great pain when he attempted it; but was quite easy in bed when lying on his right side, but could not lie on his left side. His pulse, for near two months before his death, was quite regular, though before that time it had been otherwise, and the apothecary who had attended him had suspicions of hydrothorax. There was a swelling in the abdomen, which was very painful to him. This proved to be a cancerous tumour of the whole of the omentum, which, being very heavy, when he attempted to get up gave him the pain mentioned before."

"Mr. Cruickshank said that he saw a case of hydrothorax where there was no pulsation to be felt, either in the carotids, or in the arteries at the wrist, or in the groin, nor could any motion be perceived at the part where the heart is usually felt pulsating; and the patient continued in this state for two months."

Dr. Physick continued to prosecute his studies with the most examplary perseverance and industry, under the immediate superintendence of Mr. Hunter, throughout the year 1789. On the first of January, 1790, he was appointed House Surgeon to St. George's Hospital for one year, which is the usual period of that service in the institution. This appointment he owed exclusively to the patronage and influence of Mr. Hunter. The advantages offered by such a situation to the student of medicine, in the way of promoting and facilitating his acquisition of practical knowledge and skill, were of the most important character; and consequently they were much too well known and appreciated not to cause the place to be sought after by numerous applicants, most of whom, from the circumstance of their English birth alone, it might be supposed, could have exerted an influence more powerful than that of a foreigner. Here were exemplified in the most happy manner, the important advantages which Dr. Physick derived from the favourable impressions which Mr. Hunter had imbibed respecting his general worth, his talents, and his acquirements. These considerations induced him unhesitatingly to exert the whole of his influence in behalf of Dr. Physick, who accordingly succeeded over all his competitors. A few months after this period, Dr. Physick had an attack of indisposition, which was of so severe a character that Mr. Hunter became very uneasy and alarmed about him, and was on the eve of insisting upon his return to America. This attack, I have no doubt, was principally owing to the laborious life which he led, and the close confinement to which he subjected himself. Providence, however, for its own wise and beneficent purposes, thought proper to restore him to health, to the great delight and gratitude of his parents and friends.

That it was during the period of his remaining in St. George's Hospital that Dr. Physick acquired a vast deal of that surgical skill and dexterity which laid the foundation of his subsequent greatness, cannot, I think, for a moment be questioned. Having his whole time occupied in administering to the wants of such unhappy objects as were suffering from the effects of accidents or disease; being constantly engaged in applying the necessary bandages and dressings to fractured bones, dislocations, wounds, and injuries of every description, and seizing hold, as was his invariable custom, of every such opportunity and occasion of making himself minutely acquainted with the best and most perfect manner of performing these services, he soon became remarkably expert in all his manipulations, and acquired a degree of experience which increased greatly his stock of practical knowledge. I think it will probably not be denied, that Dr. Physick exhibited as great a degree of neatness and dexterity in the application of bandages and dressings as any other surgeon who ever lived.

During the period of his services in this institution, he learned also the manner of constructing and contriving several kinds of instruments and apparatus, which he subsequently was the first to introduce into this country, to the great benefit of our art.

An anecdote frequently related to me by Dr. Physick, connected with his early appointment to St. George's Hospital, I trust I may be pardoned for mentioning here, notwithstanding it has already been promulgated from another source. His success in obtaining this situation caused some slight degree of dissatisfaction on the part of some of the disappointed applicants, who conceived that their claims for the situation were stronger than his. In consequence of this Dr. Physick clearly perceived that they evinced an uncommon share of curiosity respecting the manner in which he discharged his duties, and that they were disposed to scrutinize his actions with the greatest strictness. A short period after commencing his services, a patient was admitted into the hospital who had had the misfortune to dislocate his shoulder; the head of the humerus was thrown downward, and lodged in the axilla. Fortunately the accident was quite recent. It so happened that at the time the man was admitted the whole class were in attendance at the house. They, of course, were exceedingly anxious to witness the manner in which the reduction would be effected, and Dr. Physick was perfectly well aware that his method of restoring the bone to its natural situation would be subjected to severe criticism. He directed the patient to be seated upon a high chair; he then proceeded to examine the injured shoulder very particularly, and questioned the man as to the manner in which the accident had occurred. Whilst making these inquiries he placed his left hand in the axilla, and taking hold of the lower end of the humerus with his right hand, he made all the extension in his power; he then suddenly depressed the elbow of the patient to the side of his body, and in so doing, dislodged the head of the bone, which glided instantaneously into the glenoid cavity, very much to his own delight, and doubtless also to the perfect satisfaction of the class.

In relating this incident Dr. Physick never seemed disposed to assume to himself much merit for affecting such a speedy reduction; he rather wished to communicate the impression that his success was in a great degree owing to all the circumstances of the case favouring an easy reduction. I was always of the opinion, however, that his characteristic modesty induced him to underrate his services; and that his success in reducing the dislocation so speedily, unaided, was principally owing to that unrivalled address and dexterity of which he subsequently proved himself to be so completely a master. The treatment of this case produced the most happy influence in promoting the interests and comfort of the Doctor during the remainder of his stay in the hospital. He stated that from that time forward he always enjoyed the uninterrupted regard and respect of the medical class.

In January, 1791, the period for which he had been elected to St. George's Hospital having expired, he quitted the institution, carrying with him the warmest testimonials from its proper authorities, of his most excellent medical qualifications, and also of his general good conduct. They went so far as to declare, that instead of considering him to lie under any obligations to the institution, they considered the institution indebted to him for the many benefits he had conferred upon its unhappy inmates, and for the useful results which had been produced by the employment of his singular zeal and abilities. He now received his diploma from the Royal College of Surgeons in London.

Soon after leaving St. George's Hospital, Dr. Physick received from Mr. Hunter a mark of his respect and esteem, which was gratifying to him in the highest degree, and more particularly so as it furnished the most conclusive evidence of Mr. Hunter's entire confidence in his professional skill and attainments. Mr. Hunter invited him to take up his residence with him, to become an inmate of his house, and to assist him in his professional business; he also held out inducements to him to establish himself permanently in London, and to pursue the practice of his profession in that city.

Notwithstanding the tempting nature of these offers, and the great advantages which Dr. Physick might have derived from accepting them, it did not comport with either his own designs, or those of his father, that he should exile himself from his native country. He, however, gratefully accepted Mr. Hunter's offer to reside with him until the period should arrive when, in accordance with the plan previously laid down for the completion of his medical education, he was to visit Edinburgh, in order to graduate in medicine in the University of that city. In conformity with this arrangement he remained with Mr. Hunter, and rendered him every aid and assistance in his power, not only in his professional business, but also in the prosecution of his physiological experiments, and the making of anatomical preparations, until May, 1791, when he took his final leave of London. I may notice that his father had, previously to this period, returned to America.

The parting between Mr. Hunter and Dr. Physick was painful to the latter to an extreme degree, and certainly the most distressing event which occurred to him during his stay in London. The ties which bound him to Mr. Hunter were of no ordinary description. Mr. Hunter had not only extended towards him the warmest friendship and regard, but had also conferred the most invaluable benefits upon him, by giving him the advantages of his powerful aid and influence, and by promoting, by all the means in his power, his medical researches. These obligations could only be acknowledged on the part of Dr. Physick, by the most sincere and ardent devotion to his beloved preceptor. Indeed, I think I am warranted in saying, that the admiration felt for Mr. John Hunter by Dr. Physick amounted to a species of veneration. Certain it is, that he never ceased to consider him as the greatest man that ever adorned the medical profession. Could his honoured master have been permitted to witness the closing career of his pupil, he would have felt himself amply recompensed by the harvest of fame and usefulness which the latter had gathered, in consequence of his valuable aid and instructions.

Immediately after his arrival in Edinburgh, Dr. Physick entered with his usual ardour upon the prosecution of his studies. He attended very diligently the medical lectures delivered in the University, besides which he visited constantly the Royal Infirmary, and was a careful observer of the practice pursued in that institution, and witnessed all the operations which were there performed. In May, 1792, having complied with all the requisitions demanded by the University, he obtained the degree of M. D. The subject of his thesis was apoplexy; and in compliance with the established regulations he was obliged to write it in the Latin language. The original manuscript copy of this essay, which he first wrote in English, is now in my possession, and it bears the most satisfactory evidence of having been prepared with a vast deal of careful attention. It is divided into distinct chapters, and contains particular memoranda of the several authors to whom he wished to refer.

In order to show the familiar knowledge of the Latin language which Dr. Physick possessed, I may relate the following anecdote. It is well known that the examinations for a medical degree in Edinburgh are conducted in Latin; and that there are many applicants for the honor who do not possess a sufficient knowledge of that language to enable them adequately to make replies from their own resources. In order to obviate this deficiency, there existed in Edinburgh a class of men termed *grinders*, whose occupation consisted in preparing students, by a system of drilling which should render them competent to reply to such questions as in all probability they would receive. It so happened that, a short time previous to the examinations, Dr. Physick was in company with a fellow-student from this city, and in reply to some allusion made by his companion to these grinders, the Doctor stated that he should not seek their aid, but that he was determined to rely upon his own knowledge of the language to carry him safely through. His companion expressed much surprise at this statement, seeming to consider it as a vain boast on the part of Dr. Physick; and he intimated his doubts of the Doctor's capabilities very clearly by the query: Do you mean to say that you possess a sufficient knowledge of the Latin to enable you to carry on a conversation in that language? Dr. Physick satisfied him completely, by instantly addressing him in Latin, and continuing for some time to reply to him in the same tongue. The inference to be drawn is, that his companion was in all probability at that very time under the tuition of a grinder.

Dr. Physick did not leave Edinburgh immediately after obtaining his honorary title: he remained there for a short period; and the manner in which he occupied himself may be fairly illustrated by the following extract, which I take from his note book.

"June, 1792.—Prepared for the house surgeon at the Royal Infirmary, Edinburgh, an intussusceptio, in which the ileum had passed into the colon, and at last dragged down six inches of the colon. Most probably there was a stricture formed about the termination of the ileum, near to the valve, as there were strictures in other parts of the intestines. At present a stricture of the ileum at this part certainly exists, but whether that did not arise from the binding of the inverted colon, and the inflammation consequent thereon, I cannot be sure. I was not present at the dissection of the body, and the person who took out the parts tore them very much."

Dr. Physick returned to his native country in September, 1792; and commenced the practice of his profession in Philadelphia. His office was situated in Mulberry street near Third. That Dr. Physick entered upon his practical career under the most favourable circumstances will, I think, be readily admitted. I have already shown that, in addition to his own extraordinary qualifications, he had enjoyed the most ample opportunities of acquiring knowledge from sources distinguished alike for their exalted character and superior excellence. Nature also rendered her best aid for fitting him pre-eminently, by all external advantages, for the successful accomplishment of his objects. His personal appearance was commanding in the extreme. He was of a medium height; his countenance was noble and expressive; he had a large Roman nose; his mouth was beautifully formed, the lips somewhat thin, and he had a high forehead, and a fine hazel eye, which was keen and penetrating. The expression of his countenance was grave and dignified, yet often inclined to melancholy, more especially when he was engaged in deep thought, or in performing an important and critical operation. Dr. Physick rarely indulged in excessive mirth; he was, however, far from being insensible to playful humour, and on such occasions his countenance would be lighted up by a benign smile, which altered entirely the whole expression of his features. His manners and address were exceedingly dignified, yet polished and affable in the extreme; and when he was engaged in attendance upon a critical case, or in a surgical operation, there was a degree of tenderness, and at the same time a confidence in his manner, which could not fail to sooth the feelings and allay the fears of the most timid and sensitive.

The introduction of a young practitioner of medicine to the notice of the community, is proverbially slow; and not unfrequently, before he can inspire a sufficient degree of confidence to lead to his employment, a length of time is requisite which, in some instances, produces bitter disappointment, and occasionally even utter hopelessness and despair. As might have been anticipated there were but few professional calls made upon Dr. Physick during the period of the first year after he had established himself in this city; and it is highly probable that, notwithstanding all the advantages of which he could boast, he would have been obliged to exercise an enduring degree of patience for a considerably longer period, were it not that in the summer of 1793, Philadelphia had the misfortune to be visited with that awful calamity, the yellow fever. It is not necessary in this place to give an account of the destructive ravages caused by this epidemic. The most ample and detailed description of the origin and progress of the yellow fever, with all its concomitant circumstances, has been furnished from the very highest source—by one of the brightest luminaries of the age; one who was a most prominent and efficient actor in the tragical scene; one whose disinterested patriotism, brilliant imagination and splendid acquirements endeared him to the hearts of his countrymen, and one also who invariably evinced himself the warm and constant friend of Dr. Physick. Need I add the name of Dr. Benjamin Rush?

The occurrence of the yellow fever afforded to Dr. Physick his first opportunity of proving to his fellow citizens his entire devotion to his professional pursuits, his utter disregard of all personal considerations which might interfere with the discharge of his duties, and the fearless intrepidity with which he exposed himself to danger, in order to contribute to the safety of others. As a means of preventing an extension of the disorder, by removing, as far as possible, from overcrowded situations such as were attacked by it, and in order also to afford an asylum and the most efficient treatment to such as were destitute, the Board of Health, in August, 1793, established the yellow fever hospital at Bush Hill, and Dr. Physick having offered his services, was elected by them physician to the institution. He immediately proceeded to the performance of his duties with the most singular ardour and ability; and during the time he remained in the hospital rendered services which were acknowledged to be of the most important character, and which served to secure to himself the approbation and esteem of the community at large. Dr. Physick did not himself escape without an attack of the fever. It however yielded to the treatment employed, although I heard him declare but a short time previous to his death, that he did not think his constitution had ever completely recovered from the shock which it then received.

During a period of such general distress history has at all times shown that the minds of the people are very apt to become excited and inflamed; and some threatening indications of riotous conduct having been exhibited whilst Dr. Physick was serving in the Bush Hill Hospital, he was created an alderman by the Governor of the State of Pennsylvania, for the purpose of enabling him to quell them.

The notoriety which Dr. Physick obtained, together with the favourable impression which he produced during his residence in the hospital, enabled him to form associations which subsequently proved of immense benefit to him in promoting his professional success. Among others, I may mention that our late fellow citizen, Stephen Girard, who was at that melancholy epoch a member of the Board of Health, and who rendered the most important services throughout the epidemic, in alleviating the miseries and providing for the wants of the unhappy sufferers, conceived a lasting friendship for him. Stephen Girard subsequently became distinguished for the acquisition of immense wealth, far beyond that of any other person in our city. Whilst living, he performed many acts of benevolence and charity, and in dying, he made a bequest which will cause his name to be transmitted to the latest posterity. The services, however, rendered by Stephen Girard, during the prevalence of the yellow fever, should never be forgotten; services which went to solace the woes of the widow and the orphan, and which form a still higher claim to the veneration of his countrymen.

Mr. Girard was well known to have been a man of very eccentric habits and strong prejudices. One of his peculiarities consisted in a general dislike of physicians. I always felt assured that this prejudice was founded upon his ignorance, and a vain belief that he knew as well from his own experience how to treat diseases, as most men who were regularly educated to the profession. He, however, made a few exceptions; and one of these was Dr. Physick, to whom he always resorted for medical advice, and assistance, whenever he deemed the case critical, as long as he lived. Mr. Girard finally died a victim to his prejudices; he was attacked with an inflammation of his chest, and would not consent to lose blood until it became too late.

Dr. Physick, I believe, was the first to promulgate the doctrine, which he founded upon his own observations, that the yellow fever was not contagious; he also fully coincided with Dr. Rush in the opinion that it was of domestic origin. Dr. Rush at first dissented from the doctrine of the non-contagious character of yellow fever, but subsequently became convinced of its truth and importance. During the prevalence of the epidemic, Dr. Physick, in conjunction with Dr. Cathrall, made a series of post mortem examinations and dissections, which were productive of the most important results; inasmuch as they went far, not only in elucidating the true nature of the disorder, but also in pointing out the best method of treatment. These dissections proved, in the most satisfactory manner, that the complaint possessed a highly inflammatory character, and that the stomach more particularly was the seat of great inflammation. They also established the most valuable therapeutic indications, by confirming, conclusively, the superiority of the antiphlogistic method of treatment, over that of an opposite character, which had generally been employed. It is quite apparent, from this notice of Dr. Physick's dissections of persons who had died of yellow fever, that he preceded the celebrated Broussais in pointing out the intimate relations which subsist between the condition of the stomach and the production of bilious and yellow fevers. It is well known, that as far back as the period to which we are alluding, Dr. Physick pronounced yellow fever to be gastritis; and he was so much influenced by his opinions of the necessity of avoiding all causes which could prolong or excite the gastric irritation, that in one instance he ascribed the death of a patient labouring under this malady, to a relapse, produced by swallowing a small quantity of chicken water.

After leaving the hospital, he removed to the city, and gave his undivided attention to his professional engagements. In the year 1794, Dr. Physick was elected, by the managers of the Pennsylvania Hospital, one of the surgeons to that institution. This period may be stated to be the dawn of his great surgical fame and usefulness. The reputation sustained by the Pennsylvania Hospital, for a long series of years, not only on account of the amount of benefits which it has conferred, but also on account of its excellent administration, are so well known as to render superfluous any encomiastic notice of it on my part. That Dr. Physick contributed largely to the support of its character and reputation, can be readily shown by a record of his services. It must be admitted, however, that his appointment to the hospital had a considerable influence in promoting his success, and leading to an extension of his business. The situation enabled him to add greatly to his stock of experience, and he also enjoyed from it the most ample opportunities of perfecting himself in the operative department of his profession. I have already stated that in his manual procedures he exhibited the utmost degree of neatness and dexterity. Dr. Physick possessed, pre-eminently, all the qualifications requisite for a bold and successful operator. His sight was remarkably keen and good; his nerves, when braced for an operation, were firm and immovable; his judgement was clear and comprehensive, and his resolutions once formed, were rarely swerved from. In addition to these, he owed much to his thoughtful and contemplative cast of character, which induced him to deliberate and reflect intensely upon all the circumstances of his case, and to make elaborately, beforehand, every preparation which might become needful in the performance of his task.

In order to appreciate fully and correctly the amount of contribution made by Dr. Physick to the department of surgery, it is important to keep in view what was the imperfect condition of the art in this country, at the period of his commencing his professional career. It is well known that the principles of science which should govern the treatment of many disorders were in that day very imperfectly understood. It is true that there were some members of the profession, endowed with great merits and learning, who devoted themselves especially to the cultivation of surgery. These gentlemen were quite competent to the performance of what were then considered the capital operations in surgery; still it must be confessed that not any one of them ever acquired the necessary degree of skill and pre-eminence to create an unlimited confidence in his abilities. In consequence of this there was no head, no rallying point to surgery, an appeal to which when once made, should be regarded as decisive. We cannot feel surprised at the comparatively insignificant position which the science of surgery then held, when we reflect that, prior to the appointment of Dr. Physick, surgery was not taught in this city as a separate and distinct department. The professorships of anatomy and surgery were combined in the University of Pennsylvania, and the duty of teaching both branches devolved upon one individual. Under these circumstances it would have been extremely unreasonable to expect an efficient course of instruction, when it is well known that the usual period allotted to a course of lectures, upon either department, as now separated, is confessedly too limited.

Soon after Dr. Physick's appointment to the Pennsylvania Hospital, his mind became considerably engaged in the consideration of a class of disorders of which that institution then had, and continues to have at the present time, its full proportion, namely, ulcers. It is undeniable that the treatment of these affections was in that day but little understood by our surgeons. The method of cure which they resorted to was for the most part exclusively empirical. They were not generally guided by correct scientific principles. In consequence of this the treatment of ulcers was notoriously unsuccessful; and I am sorry to say, that there are good reasons for believing that limbs affected with ulcers were not unfrequently amputated, owing to their ignorance and inability to accomplish a cure, which, under different circumstances, by means of judicious and skilful treatment, might have been completely restored.

Dr. Physick devoted himself in an especial manner to ameliorating the condition of this class of the patients. He laboured most assiduously in establishing a more correct and efficient method of treatment; and in a short time the results of his practice were so evidently successful as to add not a little to his rising fame and greatness. I have been told that at a very limited period after commencing his services, he had almost entirely cleared the wards of the patients affected with ulcers. His method of treatment in cases of inflamed and irritable ulcers, was exceedingly simple. He directed the patient to be confined to bed, and to be kept strictly at rest; and in cases where the ulcer was situated upon a lower extremity, he caused the limb to be considerably elevated. He next directed that mild and soothing applications should be made to the ulcer itself; and in conjunction with this he made use of proper constitutional treatment. In cases where the ulcer partook of an indolent nature, he always preferred effecting the necessary stimulation by means of local applications, whilst the patient was confined to bed, to permitting him to walk about, as sometimes recommended.

During the period of Dr. Physick's services in the Pennsylvania Hospital, he made several valuable modifications and improvements in the treatment of fractures. Without entering minutely into the consideration of these, I may refer to his modification of the celebrated Desault's apparatus for the treatment of fractures of the thigh. By increasing the length of Desault's splint, Dr. Physick has accomplished a most important object, in causing the counter-extension to be made more completely in the direction of the axis of the limb, and also in keeping the patient more strictly at rest. This apparatus of Desault, thus modified by Dr. Physick, and with the block attached to the lower extremity of the splint by Dr. Hutchinson, for the purpose of making the extension in the direction of the limb, has been used in the hospital for a long series of years, with the happiest results. Dr. Physick never ceased to regard it as the most complete and successful method of treating fractures of the thigh ever invented.

Fractures of the humerus occurring at or near the condyles, are exceedingly apt to be followed by a very unpleasant projection of the elbow. In some instances the deformity is so great as to give rise to most disagreeable consequences, more especially in case the accident should happen to a young female. To Dr. Physick is due the credit of having invented a method of treatment which has succeeded in many instances in effecting a complete cure, without the occurrence of any deformity. This treatment consists in applying to the injured limb two angular splints, which should extend from near the shoulder down to the extremities of the fingers. In addition to this he directs the patient to be kept in bed, "with the arm flexed at the elbow, and lying on its outside with the angular splints, supported by a pillow."

In cases of fracture of the lower end of the fibula, where the accident is accompanied with dislocation of the foot outward, Dr. Physick was in the habit, many years since, of treating the fracture upon a plan precisely similar to that recommended by Baron Dupuytren. To which of these gentlemen is due the priority of the invention, I am unable to say.

In the treatment of dislocations, the highest commendation is due to Dr. Physick, for being the first to carry into full effect a plan of treatment which, although originally suggested by Doctor Alexander Munro, of Edinburgh, was never put into execution, as far as we can learn, prior to its employment by Dr. Physick. I allude to the use of copious blood-letting, carried, when necessary, even ad deliquium animi, in order to produce a complete relaxation of the muscular system, and thereby facilitate the reduction of the dislocated bone. We are infinitely indebted to Dr. Physick for having directed our attention to this method of treatment, by means of which, in very many instances, old and difficult dislocations have been reduced, and limbs restored to usefulness, which otherwise would have been irrecoverably ruined.

In the year 1794, Dr. Physick was elected one of the physicians to the Philadelphia Dispensary: he visited the sick of his particular district, and performed his duties with the strictest fidelity during the period he held this appointment. He subsequently was chosen one of the consulting surgeons to this same institution, and retained the situation till the time of his death.

From a reference to Dr. Physick's papers, it appears that his professional engagements increased very considerably in the year 1795. About this period, it may be stated, that his prospects of establishing himself successfully in practice became exceedingly flattering. During the year 1795, he commenced keeping a journal of the most remarkable and interesting cases which occurred in his practice, more especially such as possessed a surgical character. This journal is continued up to the year 1810, although in consequence of the multiplicity of his engagements about this period, we have to regret, the number of cases inserted is very considerably lessened. The first case recorded in the note book, is that of a lady affected with blindness from cataract. In this case, he performed the operation of extraction of the opaque crystalline lens, with complete success, and restored his patient to sight.

I may mention here that Dr. Physick's favourite operation for cataract was that of extraction. He gave it a decided preference over all the other operations, and always performed it whenever the condition of the eye was suitable. He acquired such a perfect degree of skill in extracting the lens, that his operations were almost invariably followed by success. I am of the opinion that his operations upon the eye, in conjunction with those for stone in the bladder, did as much in establishing Dr. Physick's great surgical character as any others which he ever performed. Operations of this nature, when successfully executed in that day, were widely circulated. His first operation of lithotomy was not performed, however, until the year 1797. He subsequently performed it, as is well known, in innumerable instances, with the most extraordinary facility and success. In performing his first operation of lithotomy, he accidentally divided with his gorget the internal pudic artery. The hæmorrhage from the wounded vessel was exceedingly profuse. He immediately compressed the trunk of the artery with the fore finger of his left hand, and then passed the point of a tenaculum under it; a ligature was then cast round it and firmly tied. This of course arrested the hæmorrhage, but the ligature included along with the artery a considerable portion of the adjacent flesh. In order to obviate this inconvevience, Dr. Physick subsequently contrived his celebrated forceps and needle for the purpose of carrying a ligature under the pudic artery. Since that period this instrument has been in general use for the purpose of securing deep-seated vessels. It has twice been successfully employed in performing the operation of tying the external iliac artery; in the first instance by the late lamented Doctor Dorsey, a favourite nephew of Dr. Physick's, and one to whom he was ardently attached, and in the second instance by myself. No higher commendation could be bestowed upon this instrument than may be inferred from the numerous modifications which have since been made of it. I must be permitted to declare, that in my opinion, the original instrument, as designed by Dr. Physick, has never been excelled, either in point of ingenuity or utility.

In order to facilitate the division of the prostate gland and neck of the bladder, in performing the operation of lithotomy by means of the gorget, Dr. Physick suggested a valuable improvement to the instrument as used by Mr. Cline which has since been almost universally employed in this country, and has received the entire sanction and approbation of our most distinguished surgeons. A full description of Dr. Physick's gorget was published in the first volume of Coxe's "Medical Museum," for the year 1804, by Mr. R. Bishop, a surgeons' instrument maker of high repute, then living in this city. It is also noticed at length in Dr. Dorsey's "Elements of Surgery." The modification consists in having the great gorget so constructed, that a perfectly keen edge may be given to that part of the blade which commences the incision, and which is connected to the beak of the instrument. For this purpose the beak and blade are separable, and so arranged that the blade may be connected to the stem and firmly secured by a screw. Without this arrangement it is exceedingly difficult to impart a fine edge to that part of the blade which is contiguous to the beak, and inasmuch as the incision of the neck of the bladder is commenced at that point, the success of the operation must necessarily be much influenced by it.

During Dr. Physick's attendance at the Pennsylvania Hospital, in the year 1796, a case occurred in which the patient, a young man, had laboured under a suppression of urine for forty-eight hours. The bladder was so much distended that it rose above the umbilicus, and the patient was suffering intense agony. Dr. Physick made repeated attempts to introduce catheters of different sizes into the bladder, in order to draw off the urine, but without success. He next took a bougie and succeeded in introducing it into the bladder, but upon withdrawing the instrument, no urine followed. The idea then struck him that he might fasten the point of a bougie upon the extremity of an elastic catheter, so as to conduct the catheter into the bladder and allow the urine to flow through it. He immediately carried his plan into execution, and succeeded most happily in completely relieving his patient. Since then this method has been frequently resorted to with great success, in cases where, owing to enlargements of the prostate gland, strictures of the urethra, and other causes, the common catheter could not be passed into the bladder. Dr. Physick communicated an account of this case to Dr. Miller, which is published in the New York Medical Repository, vol. vii. p. 35, together with this method of preparing the intrument, and some experiments on the treatment of gum elastic by spirit of turpentine and ether; describing also a method of coating catheters with gum elastic. A full description of the bougie-pointed catheter is also given in Dorsey's Elements of Surgery.

In the treatment of strictures in the urethra, Dr. Physick displayed the most enviable degree of skill. It is true that he made the management of this disorder a very particular study, and the tact and dexterity which he exhibited in dilating a stricture, was sufficient to excite the warmest admiration. What department of surgery was there which was not in some way or other enriched by his labours? Among his other contributions, however, let us notice his invention of an intrument, in the year 1795, for the purpose of cutting through a stricture which had refused to yield to the ordinary methods of treatment. This instrument consists in a lancet concealed in a canula, which is passed down to the stricture, and then the lancet is pushed forward so as to effect its division. After the stricture is cut through, a catheter or bougie should be introduced and worn for some time, in order to produce the necessary permanent dilatation. The success attending this method of treating strictures, which have resisted all other attempts at dilatation, has now become so completely familiar, as to entitle it to be considered one of the most important and useful operations in surgery. It should be stated also, that in some cases of complete retention of urine from stricture of the urethra, this method of dividing the stricture by means of the lancet has obviated the necessity of puncturing the bladder itself.

If I mistake not, Dr. Physick was the first who pointed out to our surgeons the method of constructing the waxed linen bougie. He informed me that soon aft r his return from Europe he was engaged in attendance upon a patient, in conjunction with his much esteemed friend Dr. Wistar. It so happened that in the treatment of this case there was occasion for a bougie of a particular size and shape. Dr. Wistar regretted very much that he did not possess such an instrument, and he expressed his doubts as to whether they would be able to procure one. Dr. Physick told him that he need not be uneasy, for that he would furnish the instrument; and accordingly he went to work and constructed one himself of the precise kind which they wanted, to the great surprise and gratification of Dr. Wistar.

I may mention that in the treatment of strictures of the urethra, Dr. Physick invariably preferred using waxed linen bougies of his own make to either the metallic or imported gum elastic bougies. He considered his own to be much the most safe and valuable. It is proper, however, to state that the gum elastic bougies which were imported into this country in that day, were of a very inferior quality to those which we now have. It is more than probable that even at the present time there are but few surgeons who understand the method of properly preparing the waxed linen bougies, or who would take the trouble to make them even if they were acquainted with the method. I do not hesitate to assert, however, that from long practice and dexterity, Dr. Physick acquired the art of making a more beautiful and perfect instrument of this kind than any other man living. A general account of the method of preparing the waxed linen bougies is contained in "Dorsey's Elements of Surgery."

During the years 1797, 1798, and 1799, the yellow fever again broke out in our city, and Dr. Physick was again found in the foremost rank of those who had to contend against its ravages. Whilst engaged in the performance of his duties, in the year 1797, he was attacked himself for the second time, with the fever, and his illness was so severe that for some time but slight hopes were entertained of his living. His recovery from this indisposition was exceedingly slow, and he was left in such an enfeebled state that he was advised by his medical friends to make an excursion into the country in order to recruit his strength. He accordingly took this opportunity of paying a visit to his brother, who was living upon a beautiful farm, situated on the banks of the Susquehannah, in Cecil county, Maryland. He was somewhat amused, whilst performing this journey, at being informed, by an innkeeper on the road, that Dr. Physick, of Philadelphia, was dead. His health was greatly benefited during the period of his sojourn with his brother, and it appears that he conceived a warm attachment to the place, inasmuch as after the death of his brother, many years subsequently, he became the purchaser of the estate, and during the latter years of his life he was accustomed to spend a part of each of the summers upon it.

During the prevalence of the yellow fever in 1798, Dr. Physick was again resident physician at the Bush Hill Hospital; and upon leaving the institution after the subsidence of the epidemic, he was presented in a flattering manner, by the board of managers, with some valuable silver plate, as an acknowledgment of their "respectful approbation of his voluntary and inestimable services."

In the winter of 1798, Dr. Physick read a paper before the "Academy of Medicine of Philadelphia," containing "Some Experiments and Observations on the mode of operation of Mercury on the body." This paper was subsequently published in the New York Medical Repository, vol. v., p. 288. The result of these experiments and observations goes to disprove the opinion that the different preparations of mercury produce their effects on the system, in consequence of their being absorbed and carried into the blood. The experiments made by Dr. Physick in order to detect the presence of mercury in the blood and saliva of patients undergoing salivation from that article, were repeated by Dr. Seybert, but both were unable to discover the presence of the metal.

I have already stated, that in consequence of the untiring zeal of Dr. Physick in investigating the nature and phenomena of the yellow fever, aided by the ample opportunities which he enjoyed of prosecuting his researches, he was led to the adoption of some views which were not only of an interesting and novel character, but such also as had a most important bearing in elucidating the true pathology of the disease, and in establishing in consequence more correct therapeutic indications. It was after the subsidence of the epidemic of 1799 that he published in the New York Medical Repository, "Some Observations on the Black Vomit." In this communication he relates a series of careful and well conducted experiments, which prove most conclusively that the matter of black vomit, so far from being poured out by the vessels of the liver, as was the commonly received opinion, is produced by a secretion from the inflamed vessels of the stomach and intestines. These observations, showing that the effusion of black vomit must be regarded as one of the modes in which violent inflammation of the stomach has a disposition to terminate, not only went far in destroying the preconceived notions entertained by many physicians, that the yellow fever was a disease of debility, and that the black vomit was to be regarded as a putrid phenomenon, but also confirmed, most satisfactorily, the propriety of the antiphlogistic method of treatment.

The year 1800 formed a most eventful one in the life of Dr. Physick. During this year he formed a matrimonial alliance with Miss Elizabeth Emlin, a highly gifted and talented lady, and daughter of one of the most distinguished ministers of the Society of Friends. By this marriage he had four children, two sons and two daughters, all of whom are now living.

In the year 1800, a request was made to Dr. Physick in writing, by a number of gentlemen engaged in attending the medical lectures delivered in the University of Pennsylvania, that he should lecture to them on surgery. Among these gentlemen who so fully appreciated his extraordinary qualifications, was included our present pre-eminently distinguished Professor of the Theory and Practice of Medicine, Dr. Chapman.

No man could feel more deeply the solemn responsibilities attendant upon such an enterprise, than Dr. Physick. After mature deliberation, however, he determined to accede to their request, and this may be dated as the period at which he commenced his labours as a lecturer.

The following anecdote, related to me by the Doctor himself, will exemplify the ardour and zeal with which he entered upon the performance of his duties, and it illustrates, also, most happily, the great advantages which may be derived from a word of encouragement and approbation, coming from a source in which entire confidence is reposed.

After preparing the lecture introductory to his course, he committed it to memory. Among the persons invited to be present at its delivery, was his valued friend, Dr. Rush. The scene was a trying one to Dr. Physick. It was the first time he had ever publicly addressed an audience. I have been informed, however, that he acquitted himself extremely well. At the close of the lecture, Dr. Rush stepped up to him and gave him his hand, and congratulated him upon his success. He then said to him very emphatically, "Doctor, that will do—that will do. You need not be apprehensive as to the result of your lecturing—I am sure you will succeed." Dr. Physick never forgot Dr. Rush's kind manner to him on this occasion. He assured me that it exerted a considerable influence in strengthening and confirming his resolutions to persevere. It is needless for me to say that Dr. Rush's predictions respecting Dr. Physick's ultimate success in lecturing, were fulfilled to the utmost. Five years subsequently to that period, the Professorship of Surgery was created in the University of Pennsylvania, and Dr. Physick was appointed to fill the chair.

In the year 1801, Dr. Physick received an appointment to the Philadelphia Alms House Infirmary. In looking over his papers a short time since, I discovered the letter which notified him of his election. I am induced to insert the letter in this memoir, inasmuch as it is somewhat peculiar, and the terms in which it is worded, evidently admit of the construction, that his appointment carried with it unusual powers and privileges.

"*Alms House*, 16*th Sept.*, 1801.

"Esteemed Friend—I take the liberty to inform you that, by a Resolution of the Board of Managers of the 7th inst., you were appointed *Surgeon Extraordinary*, and on the 14th following, one of the Physicians of this Institution; and with sentiments of sincere regard

I remain your friend,

Peter Browne.

Dr. Philip Syng Physick.

I am not aware that any similar appointment has been made in that institution since that period up to the present time. I remember distinctly that he informed me on several occasions, that he held the situation of *Surgeon Extraordinary* to the Alms House Infirmary.

In 1802, he published a paper in the New York Medical Repository, in which he communicates the particulars of a case of hydrophobia. In this communication he gives a circumstantial account of the appearances which were observed upon dissection; and as a means of affording relief in similar cases, he suggests, in conjunction with other remedies, the propriety of performing the operation of tracheotomy. The following quotation is sufficiently explanatory of the views which he entertained.

"Reflecting on the symptoms which took place in the case above related, it appeared to me, that the dread of water arose chiefly from the convulsive or spasmodic contraction of the muscles of the glottis, which rendered the patient unable to breathe, and involved him in all the horrors of impending suffocation. When asked why he could not drink, he answered, that whenever he attempted to swallow any thing it took his breatn away."

"Under the influence of these opinions I am disposed to believe, that tracheotomy would have saved my patient, at least for a time, if it had not altogether prevented the fatal termination of the disease. I cannot suppose that the spasms of the muscles in hydrophobia would be attended with much danger to life, were it not for their influence in suspending respiration." * * * * * *

I am not informed that he ever had an opportunity of testing practically the value of the foregoing suggestion, by the performance of the operation.

About this period, it may be said, that the talents and acquirements displayed by Dr. Physick began to be extensively known and appreciated, not only by the members of his own profession, but also by those who cultivated science in general. I may mention, that in this same year, (1802,) he was elected a member of the American Philosophical Society, a well merited tribute due to his rising greatness.

The year 1802 was also signalised by Dr. Physick by his invention and execution of an operation which not only forms one of the most brilliant achievements of mordern surgery, but has also been productive of the most beneficial results to suffering humanity. On the 18th of December, 1802, he performed, in the Pennsylvania Hospital, his celebrated operation in passing a seton between the ends of an ununited fractured humerus, for the purpose of causing a deposition of callus, and thereby producing the consolidation of the broken bone. The patient was a seaman, who had had the misfortune to fracture his left arm, eighteen months previously, whilst at sea; and in consequence of the bones not having united, the limb was rendered nearly useless. At the expiration of five months after the performance of the operation he was discharged from the Hospital perfectly cured, his arm being as strong as it ever was. Dr. Physick published an account of this case in the Medical Repository of New York, vol. i., 1804; and it was republished entire in the Medico-Chirurgical Transactions of London, vol. v., 1819.

It so happened that, in the year 1830, I was requested to visit a patient in Third street, who was lying dangerously ill from an attack of remitting fever of a high grade. A few days after my first visit, in riding past his door in company with Dr. Physick, feeling very uneasy about the condition of my patient, I requested the Doctor to step into the house and see him with me, and give me the benefit of his advice. He complied with my request, and upon entering the sick man's chamber he immediately recognised him as the individual upon whom he had performed the operation which I have just described, twenty-eight years previously. Upon questioning the patient he informed us that the arm which had been broken was quite as strong as his other arm, and that he had never sustained any inconvenience from the operation. Eventually the man died; and having obtained permission to make a post mortem examination, I procured his humerus, and still have it in my possession, regarding it as one of the most interesting and valuable pathological specimens extant. At the place of fracture, the two ends of the bone are perfectly consolidated by a considerable mass of osseous matter, in the centre of which there is a hole, showing the place through which the seton passed.

Since the performance of Dr. Physick's first operation in 1802, this method has been resorted to with entire success in numerous instances by himself and other surgeons, in effecting a cure of ununited fractures, occurring not only in the humerus, but also in some of the other bones. That this operation, like all others, occasionally fails in effecting the desired object, must be admitted; it is, however, generally conceded that it possesses many advantages over the method not unfrequently resorted to, of cutting down to the ends of the bone and sawing them off, as recommended by Mr. Charles White, of Manchester.

In describing that process, M. Boyer declares it to be "painful, terrifying, and of dubious event." He once performed it on account of a preternatural joint, situated in the middle of the humerus; the limb mortified, and the patient died on the sixth day. Independently of the greater hazard attending this method of operating, it is unquestionably much more painful than Dr. Physick's; and although occasionally it succeeds perfectly, in many instances it has entirely failed.

It is a matter of much surprise and regret, that Mr. William Lawrence, of London, a gentleman distinguished for brilliant talents and extensive learning, in speaking, in his surgical lectures, of the different methods of operating for the cure of ununited fractures, describes Dr. Physick's operation by means of the seton; but owing probably to a want of better information, seems disposed to undervalue, in a most extraordinary degree, the importance of Dr. Physick's operation, and limits amazingly its successful results.

In order to correct the inaccuracy of Mr. Lawrence's statement, and to do away the false impressions which it might create, and as an act of justice due to the distinguished inventor of the operation, my friend, Dr. Hays, published in his valuable periodical, the American Journal of the Medical Sciences, vol. vii. p. 267, a brief summary of numerous cases of ununited fracture successfully treated by means of the seton. The majority of these cases are published in the most celebrated of the European journals. I consider Dr. Hays' publication to be a triumphant refutation of what I believe to be Mr. Lawrence's unintentional misstatement. Dr. Physick was himself extremely gratified at the able manner in which Dr. Hays had vindicated the claims, which he considered his operation, for the cure of artificial joint by means of the seton, justly to possess.

From a reference to Dr. Physick's private journal, and from an inspection also of a note book, or book of cases, kept by his nephew, Dr. Dorsey, it is clearly evident that at the period to which we are alluding, Dr. Physick was fully occupied in attending to a most extensive and laborious practice. In Dr. Dorsey's note book are recorded the most interesting cases and operations occurring in the practice of Dr. Physick, to which he was a witness. It is exceedingly probable, however, that during that period there were but few operations performed by Dr. Physick, at which Dr. Dorsey was not present; for in some places I discover that he gives an account of important and capital operations performed almost daily by his uncle.

It has always been a subject of deep regret with the profession, that Dr. Physick should have evinced throughout his whole life such an extreme reluctance to the publication of the results of his valuable observations and experience. The loss which we have sustained in this respect is truly incalculable. What a fund of knowledge has in this manner been permitted to pass away, which might have been happily applied to ameliorating the wants and miseries of humanity. Strange as it may appear, I unhesitatingly assert, that posthumous fame was not sought after by Dr. Physick. I am well convinced, however, that in the latter years of his life, he regretted very much himself that he had not published more for the benefit of his fellow beings; but at this period his disinclination and habits had become so confirmed that it was impossible for him to change them.

From the paucity of Dr. Physick's printed communications, and their considerable value, I make no apology for the manner in which I briefly notice them. It has been necessary to collect them from various journals. I consider it the less necessary to enlarge upon them, inasmuch as I have pleasure in saying, that my friend, Dr. Benjamin Hornor Coates, is engaged in preparing an edition of Dr. Physick's works, with such commentaries on his doctrines and practices as may appear necessary.

In Coxe's Medical Museum, vol. i., for the years 1804 and 1805, I find there are published by Dr. Physick three papers, communicating cases occurring in his practice, together with practical suggestions, and by Mr. Bishop two, giving an account of improvements and modifications upon instruments made after the directions of Dr. Physick.

In the first paper Dr. Physick communicates the particulars of a case of varicose aneurism, occurring at the bend of the elbow, in consequence of the artery being wounded in the operation of venesection. In this case the artery had been punctured by the lancet being pushed into it through the vein. The blood escaped from the artery into the cellular membrane between it and the vein, and formed a large pulsating tumour, in which the particular thrill accompanying varicose aneurisms was distinctly felt. The sac formed out of the cellular tissue went on increasing in size, until it became so firm that the blood was forced from it into the vein through the puncture in its lower side, with sufficient force to distend it very considerably for two or three inches above and below the sac. The size of the forearm had much diminished, and the hand was constantly cold. At length the skin covering the swelling became so thinned that the patient's mind became very uneasy from an apprehension that it might suddenly rupture. In this state Dr. Wistar and Dr. Physick advised that an operation should be performed.

Dr. Physick proceeded in the following manner. He divided the skin and cellular membrane covering the swelling, and then dissected completely round the tumours. After this he tied the trunk of the vein above and below its enlargement; and next he tied the artery above and below the sac. He finally dissected out the whole of the parts between the ligatures, including the aneurismal sac. Upon opening the sac, its inside was found every where incrusted with bony matter; but the artery was perfectly sound and natural. In three weeks the wound healed, and the patient very soon recovered the entire use of the limb.

The second publication consists of a communication from R. B. Bishop, surgeons' instrument maker, to Dr. Coxe, describing the gorget, as constructed according to Dr. Physick's plan. I have already noticed this modification of the gorget in a former part of this memoir.

The third publication, contained in the Medical Museum, must be considered exceedingly valuable and interesting, from the circumstance of its being the first to announce to the profession a new method of treatment, suggested by Dr. Physick, for the relief of a most formidable variety of disease, and one which had previously baffled the skill of the most experienced physicians. In this communication Dr. Physick recommends the use of blisters for the purpose of arresting the progress of mortification. He states that he was induced to resort to this practice from a knowledge of blisters having been employed advantageously in curing erysipelatous inflammation; a practice which he learned from the late Dr. J. Pfeiffer.

In this paper Dr. Physick gives an account of two cases of mortification which came under his own notice, in which he applied blisters to the mortified parts with the most beneficial effects. He also publishes two letters, one addressed to him by his friend, Dr. Benjamin Rush, and the other by Dr. Church; each of whom describes a case of mortification in which he employed blisters, upon Dr. Physick's recommendation, with perfect success.

It is scarcely necessary for me to add, that since that period, blisters have been employed in a great variety of cases, for the purpose of arresting the progress of gangrene and mortification, with the most successful results. As a local remedy I believe a blister is entitled to a decided preference over all others. In order for it to be effectual, it should be large enough to cover the sound parts adjacent to the disease.

The fourth publication consists of a letter from R. B. Bishop to the editor, in which he gives a description of the curved bistoury, as improved by Dr. Physick, for the operation of fistula in ano, with a plate. This well-known instrument, thus modified by Dr. Physick, combines the advantages of both the blunt and sharp-pointed bistoury. Since the period of its invention it has been in general use, and is mostly found in the common pocket cases of instruments manufactured in this city.

In the fifth communication Dr. Physick describes the history of a case of luxation of the thigh bone forward, and the method which he employed for its reduction; and the paper is accompanied by a plate. Although this case is an exceedingly interesting one, I do not think it necessary to describe it more particularly.

I have already stated, that at the period when Dr. Physick commenced his professional career, the organization of the medical department in the University of Pennsylvania was so imperfect, that the chairs of Anatomy and Surgery were combined, and the duties of teaching both branches devolved upon one Professor. In order to remedy this acknowledged deficiency, in the year 1805, the chair of Surgery was made distinct from that of Anatomy, and Dr. Physick was elected, I believe unanimously, Professor of Surgery.

It should be borne in mind, that he had previously, in the year 1800, complied with a request, made to him by a number of gentlemen engaged in the study of medicine, that he would deliver lectures on surgery. These lectures were delivered in the Pennsylvania Hospital; and he exhibited such positive and satisfactory evidence of his fitness and entire competency to the task which he had assumed, that his labours were crowned with the most complete success, and he very soon became exceedingly popular as a teacher, and added greatly to his fame and celebrity.

It is more than probable that the position which he now held as a lecturer on surgery, excited no little influence in producing the change which was made in the medical faculty.

I presume it will not be denied that, however, great the advantages may have been which accrued to Dr. Physick in consequence of his being appointed Professor of Surgery in the Univesity of Pennsylvania, the institution itself derived equal advantages from his connection with its medical faculty. It is very certain that, soon after he was appointed Professor of Surgery, the number of students who resorted to this city to attend the medical lectures, increased to a prodigious extent; and although I freely admit that there were many co-operating circumstances present which tended to produce the same effect, and that his efforts in behalf of the school were seconded by colleagues who possessed talents of so refulgent a character that the light shed from them has not yet passed away, still it is worthy of record, that at the period when Dr. Physick enjoyed the very zenith of his fame and usefulness, the University of Pennsylvania had attained the acme of its reputation.

Having shown that Dr. Physick's efforts as a private lecturer were attended with the most entire success, we can readily believe that he was quite ready and prepared to enter upon the duties of his new appointment. Inasmuch, however, as this situation opened to him a more extensive field of action than he had previously cultivated, he felt himself called upon to make renewed exertions.

It is almost impossible to conceive of the great amount of labour which he was in the habit of performing daily, during this period of his life. He has frequently told me that it was his custom throughout the winter months, to rise at four o'clock in the morning. This hour being too early to disturb a servant, he was obliged to arrange his own fire. He would then sit down to his desk and prepare his lecture for the day; after which he would dress himself, and then take his breakfast, and leave his house between eight and nine o'clock, in order to attend to a most extensive and laborious practice. In addition to all this, he discharged his duties as Surgeon to the Pennsylvania Hospital, and to the Alms House Infirmary. He used often to remark, that in order to obtain entire success as a practitioner of medicine, it was necessary to work hard. He told me that in London this idea was conveyed by the emphatic expression, "Doctor or Mr. —— is working his way into business." It will be conceded that no portion of his success ever came to him gratuitously; on the contrary, he made laborious exertions to obtain it.

Dr. Physick's manner as a public lecturer was grave, dignified and impressive to an extraordinary degree. His style was clear and comprehensive, simple yet chaste. He was uniformly careful never to say too much. His choice of language was remarkably good, and he possessed the happy faculty of communicating knowledge agreeably and well in as great perfection as any other man I have ever heard lecture. Perhaps one great reason for this was, that he never undertook to instruct others upon subjects which he did not clearly comprehend himself. He attempted no display of oratory; neither did he permit his reason and imagination to run wild in the regions of theory and fancy. For these attributes he found much better employment; he kept them constantly occupied in studying the realities of life, and in reflecting upon the best methods of promoting the welfare of his fellow creatures. His lectures were all carefully prepared and written out. He did not at all approve of extemporaneous lecturing; as he thought that in lecturing upon scientific subjects, and more especially such as involved the lives and happiness of our fellow beings, no man had a right to place so much confidence in the strength of his memory as is implied in that practice.

Dr. Physick's course of lectures on surgery was pre-eminently valuable, in consequence of its being founded principally upon his own practical knowledge and experience, and in consequence also of his discarding all inferences drawn from hypothesis; besides which his lectures derived an additional attraction and importance from the circumstance that his reputation for stern integrity and strict veracity was so well known and established, that whenever he asserted facts to be true, they were implicitly believed.

As a letter-writer he was exceedingly exemplary and peculiar. I regret very much that I have not the privilage of inserting a few of his letters in this memoir, in order to let them speak for themselves. His letters in general were remarkably brief and pithy. Having said all that he considered necessary for the elucidation of his subject, he invariably stopped. I have frequently known him to reply to a letter of three or four pages closely written, in about as many lines. He was excessively annoyed at receiving, and being obliged to read letters of an unmeaning and unnecessary length. The same thing took place with respect to books. I have often heard him complain, that it was very hard he was obliged to read through a volume of two or three hundred pages, in order to get at ideas which might have been embodied in ten or twenty.

The year 1809 has been rendered memorable in the annals of surgery, by the invention and execution of an operation by Dr. Physick, which, for the brilliancy of its conception and the important practical results which have ensued from it, has excited admiration and attention throughout the medical world.

In the month of January, of the year 1809, Dr. Physick performed his operation for the cure of artificial anus, which, as is well known, eventuated in the most complete success. To those who are entirely unacquainted with the nature and condition of this loathsome malady, it is impossible to convey any adequate idea of the many afflicting circumstances connected with it; suffice it to say, that the unhappy sufferer is rendered disgusting, not only to himself, but also to all those around him. I imagine there are but few who would hesitate long in choosing between death and existence, complicated with a train of such insupportable evils. What an immense amount of obligation are we not under to him who, by the force of his genius and profound acquirements, was enabled to triumph over obstacles of such fearful magnitude, and provide a remedy for such a hopeless calamity! We are happy to say, that the debt of gratitude has not been left unpaid, and that Dr. Physick has received the homage of the profession for having achieved this invaluable discovery.

His method of perfoiming this operation is now so well known that it is not necessary for me to communicate the details of it here. He was negligent in not making a printed publication of the method at the moment of its discovery; he, however, publicly taught, in his surgical lectures, the manner of performing the operation, and the principles upon which it was founded, from the year 1809 until 1821, to classes of several hundred medical students.

You are aware that some years subsequently to the period when Dr. Physick first performed it, one of the most distinguished surgeons of Europe, the late Baron Dupuytren, performed an operation upon a somewhat modified plan, but with similar views, and founded upon precisely the same principles; and that he claimed the merit of having invented the method, and appropriated to himself the consequent honours. It did not, however, by any means comport with the views entertained by the surgeons of our country, that the distinguished head of the profession should be dispossessed in so unceremonious a manner, of honours exclusively his own. Accordingly, in order to place the matter in its proper light, my friend, Dr. Benjamin Hornor Coates, obtained from Dr. Physick the date of the operation, together with ample notes of the case, taken from his private journal, now in my possession, and also procured an account of the case as recorded in the manuscript case book of the Pennsylvania Hospital; and then published a full account of Dr. Physick's operation in the North American Medical and Surgical Journal for October, 1826, together with some valuable remarks upon Baron Dupuytren's method of operating, proving in the most satisfactory manner, that the justly celebrated French surgeon promulgated the idea of the operation long after Dr. Physick.

Notwithstanding, as might be supposed, Baron Dupuytren exhibited reluctance to yield his claims to this discovery, I am of the opinion, that previously to his death, he was fully satisfied that Dr. Physick preceded him in its invention.

In the year 1835, Dr. Physick was exceedingly gratified at receiving a letter from his relative, Dr. Robert R. Dorsey, then residing in Paris, in which he informed him that M. Roux, the present distinguished successor to Baron Dupuytren, as surgeon in chief to the Hôtel Dieu, stated distinctly in a lecture introductory to his clinical course on surgery, in the presence of Professor Mott, of New York, Dr. A. B. Tucker, of this city, and a large class of medical gentlemen, that to Dr. Physick was unquestionably due the honour of having invented the operation for artificial anus, which had been claimed by his predecessor, Baron Dupuytren.

In the third volume of the "Eclectic Repertory," for October, 1812, Dr. Physick published an account of a new method which he had employed for the purpose of extracting poisonous substances from the stomach. In this communication, he furnished the particulars of two very interesting cases, in which two children, twin brothers, of the age of three months, had been thrown into a state of complete stupor, from which they could not be roused, from having had administered to each of them, by their mother, one drop of laudanum, in order to allay the restlessness attendant upon whooping cough, under which they were both labouring. It appears that the vial from which the laudanum had been given, had contained, several weeks previously, nearly one ounce of that medicine; but in consequence of having been left without a cork, it evaporated away so much that the mother was only able to obtain one drop for one of the children, and in order to procure another drop, she put two drops of water into the vial, and stirred it about so as to obtain another drop, which she gave to the other child. The poor mother was entirely ignorant of the immense additional strength which the dose had gained, in consequence of the evaporation which had taken place.

Each of these children had been thrown into convulsions. When Dr. Physick arrived at the house, he immediately prescribed an emetic of ipecacuanha, and directed it to be given at once. This, however, could not be accomplished, as the children were incapable of swallowing. "The countenances of the children became livid, their breathing very laborious, with long intervals between the times of each inspiration, and the pulse in each very feeble. The pulse and respiration had almost ceased; and, indeed, the pulse could not be perceived, except a faint stroke or two, after that kind of imperfect and convulsive inspiration which is commonly observed in children just before actual death, accompanied with a convulsive action of the muscles of the mouth and neck." Under these circumstances, Dr. Physick saw clearly that in order to give the children a chance of recovery, no time was to be lost; and inasmuch as they could not swallow any thing, he determined to inject an emetic into their stomachs. For this purpose, he introduced a large flexible catheter down the œsophagus, and through it he injected one drachm of ipecacuanha mixed with water, by means of a common pewter syringe. After waiting some little time for the operation of the emetic, although in vain, as the stomach had, in both instances, completely lost its power of action, he injected a quantity of warm water, and then withdrew it, by means of the syringe. He now repeated these operations again and again, until he had washed out the stomachs thoroughly, and removed all their contents.

By the time these operations were completed, however, all signs of animation in each of the children were entirely lost. Discouraging as these circumstances were, the Doctor determined to persevere in his efforts to restore life; and accordingly, he injected into their stomachs some spirits, mixed with water, and a little vinegar; and he also made use of external stimuli. In a few minutes the pulse and respiration returned in each child, and in the course of a short time, both were regularly performed. The result of these cases was, that one child expired the next morning, and the other completely recovered.

In a note to this communication he states, that the idea of washing out the stomach in cases where large quantities of laudanum or other poisons had been swallowed, occurred to him at least twelve years previously, and that he had constantly recommended it in his lectures. He states, also, that his nephew, Dr. Dorsey, had performed the operation of washing out the stomach in such a case in the year 1809. At the time Dr. Physick made this communication, he was under the full impression that he was the earliest inventor of this operation. In the same volume, however, of the Eclectic Repertory, p. 380, there is published a letter from him, addressed to the editors, in which he says that he considers it an act of justice, to inform his medical brethren that the merit of prior invention belongs to Dr. Alexander Munro, jr., of Edinburgh, who published it in his inaugural thesis, in A. D. 1797. Dr. Physick was entirely ignorant of this fact until he saw it mentioned in Dr. Munro's work on morbid anatomy, which he had but very lately received.

Conceding to Dr. Munro all the honour arising from the discovery of this valuable method of treatment, it must be admitted that Dr. Physick is entitled to the grateful thanks of the community for having introduced it into practice. It is scarcely necessary for me to say that this operation is now so completely established, as to constitute it one of almost daily performance. It has been attended, in innumerable instances, with the most successful results; and by resorting to it, very many wretched and unhappy beings have been rescued from an untimely grave.

In the winter of 1813–14, Dr. Physick suffered from a severe attack of typhus fever. On this occasion his illness was so extreme that his medical friends despaired of his life for some time. He gradually got well, but his constitution never entirely recovered from the shock which it then received. It may be stated that from this period he never enjoyed what might be called uninterrupted health. His powers of digestion became exceedingly impaired, and from this cause ensued a train of most unpleasant dyspeptic symptoms. He became subject also to frequent attacks of catarrh, and his susceptibility to this condition increased to such an extent that he was obliged to observe the most rigid precautions in order to guard against it. His method of treatment when labouring under a severe cold, required confinement to a warm room; and in fact he accustomed himself to a degree of heat in his apartments which to many others was almost insupportable. In addition to this he always employed the strictest antiphlogistic treatment, as regarded his diet and his remedial agents. I was always of the opinion, however, that he injured himself, and in a measure produced the very enfeebled and prostrated condition of his system which attended him during the latter years of his life, by the excessively reducing system of treatment to which he had recourse.

The small amount of food of which he would sometimes permit himself to partake, is almost inconceivable; and this for many days together. I frequently expressed to him my regrets respecting the meagre diet he was using; and upon one occasion I dissented roundly from the propriety of such a course of dieting. He replied that he regretted it very much himself, and that he wished he could indulge in more generous living, but that he had accustomed his stomach for so long a time to abstinence from rich food, that it was impossible now to make any change.

About the period to which we are alluding he began to experience certain unpleasant symptoms, indicative of a deceased condition of the heart, and which eventually terminatad in organic affection of that organ, and doubtless laid the foundation for the hydropic complaint of which he died.

Among the complicated forms of disease to which he was subjected, must also be enumerated nephritic disorder, with calculous concretions in the kidneys. It is impossible for language to describe the pain and agony which he frequently endured from the passing of the small calculi through the ureters into his bladder. Upon one occasion, about ten years previous to his death, I knew him to be for near two hours without any pulse perceptible at the wrist, in consequence of intense suffering, caused by the lodgment of a small calculus in the ureter. It remained fixed in this situation for some days, and grew to the size of a small pea; it finally passed into the bladder, and was discharged a few minutes subsequently through the urethra. Had it remained in the bladder but for a short period, it might have attained a size too great to admit of its discharge through the canal; and he would then have had, in addition to his other evils, that formidable affection, the stone.

The practical knowledge and experience which Dr. Physick derived from the careful and minute attention which he bestowed not only upon every department of his profession, but also, I may say, upon each separate and individual case of disease which came under his notice, enabled him to suggest numerous modifications and improvements which have exerted the happiest influence in elevating the condition of our science. It would be impossible, in a communication of this nature, which has already exceeded the limits originally proposed, to give even a brief outline of the many valuable inventions for which we are indebted to him. In order to do this, it appears to me, that it would be necessary to review almost every professional act of his life; because there was no form of disease of which he undertook the management, in which he did not exercise a tact and method of treatment peculiarly his own. I do not mean to say that in every case he prescribed a new remedy, and one original with himself. My meaning is that he invariably modified either the dose, or the preparation, or the time of its administration, or the method of its application, according to his own proper and peculiar views.

It may not be deemed uninteresting to mention the particulars of a case in which he was instrumental in preserving the life of a valuable and distinguished lady, by the following simple treatment. This lady was brought on to Philadelphia labouring under an attack of dyspepsia of the most aggravated character. The irritability of her stomach was so great, that it had rejected every variety and form of nourishment which could be thought of, and her system consequently was so much weakened and prostrated, that she appeared to be absolutely dying of inanition. When Dr. Physick saw her, after proposing a variety of articles he asked her whether she had ever, since her attack, tried to take milk. She replied that she had often taken it, but her stomach very soon rejected it. He then asked her whether she did not think that her stomach would retain the half of one tumblerful of milk? She said, no. He repeated his questions. Would it retain one wineglassful? No! Would it retain a tablespoonful? No! He then told her that he was under the impression that she could retain in her stomach one teaspoonful of milk; and accordingly he prescribed the article for her, to be taken in that quantity at repeated intervals. The lady adopted his views, attended to his prescription, and was ultimately restored to perfect health.

Among other improvements suggested by Dr. Physick, I should mention, that in the Eclectic Repertory, vol. vi., for the year 1816, he published an account of a method which he had proposed for forming ligatures out of animal fibre. He had repeatedly noticed, that after the performance of operations, the wound was prevented from healing, and the patient was subjected to the greatest inconvenience and distress, in consequence of the ordinary ligatures, formed out of silk or flax, remaining fixed in the wound for the period sometimes of many weeks or even months. Under these circumstances, not only is the wound prevented from healing and the patient's health injured, but in order to remove the ligature by pulling at it from day to day, patients have been subjected to a degree of pain which, as they have been known to declare, exceeded that of the original operation. Dr. Physick considered it an object of extreme importance to obviate these inconveniences; and accordingly he proposed the use of animal ligatures, by means of which an artery could be secured for a sufficient length of time to cause the obliteration of the vessel, and the ligature, being decomposed and dissolved, would escape in the course of a few days.

His views upon this subject will be fully explained by the following quotation. "Several years ago, recollecting how completely leather straps spread with adhesive plaster, and applied over wounds for the purpose of keeping their sides in contact, were dissolved by the fluids discharged from the wound, it appeared to me that ligatures might be made of leather, or of some other animal substance, with which the sides of a blood-vessel could be compressed for a sufficient time to prevent hæmorrhage; that such ligatures would be dissolved after a few days, and would be evacuated with the discharge from the cavity of the wound."

From this period he continued to employ animal ligatures almost exclusively up to the time when he left off operating. I regret very much that notwithstanding the advantages which these ligatures possess, they are but seldom used by the surgeons of the present day. I can attribute this neglect of them to nothing but the slight trouble attendant upon their preparation.

Some time subsequently to Dr. Physick's publication upon this subject, it was shown that the idea of preparing ligatures from animal fibre had been suggested a long time previously by one of the older surgeons. It is scarcely necessary for me to say, that he was entirely ignorant of this fact, and that at the time he was under the full impression that the suggestion was novel when it originated with himself.

Whilst upon the subject of ligatures, it may not be amiss to give an account of a very ingenious contrivance which Dr. Physick employed for the purpose of facilitating the discharge of ligatures which remained fixed in the cavity of wounds, either in consequence of being penetrated by new granulations, or from other causes. In such cases he twisted the ligature very firmly, and then secured it to the adjacent skin, by means of a small strip of adhesive plaster. The effect of this twisting is to tighten the noose at the extremity of the ligature, so as to compress completely the parts contained within it; and in addition to this, the natural tendency of the ligature to untwist itself keeps up a constant action and pressure upon the parts, and thereby causes ulceration. We have known several instances in which ligatures which had been retained for a long period in wounds, have been extricated by resorting to this simple process. I may state that Dr. Physick had strong objections to the use of silk ligatures, and in cases where he did not employ animal ones, he invariably preferred those made of flaxen thread or bobbin. He was of the opinion that silk ligatures were more apt to slip.

It is my impression that the period which we are now commemorating may be considered as that at which his professional engagements had acquired their greatest extent. His pre-eminence, both as a physician and a surgeon, was at that time so generally conceded in this city, as to lead to the greatest demand for his professional services. In addition to this, his surpassing fame and reputation were so completely established and so widely disseminated, as to induce strangers from all parts of our country to resort to Philadelphia, in order to be benefited by his skill and experience.

It follows, also, as a natural consequence of his exalted position, that many persons who could not make it convenient to leave their homes, would apply to him for his advice and opinions in writing; so that in addition to his other labours, much of his time was occupied in keeping up an extensive correspondence.

I have already shown that his health was considerably impaired; and it is probable that about this period he must have been deeply sensible of his increasing infirmities, inasmuch as he thought proper, in 1816, to resign his situation as Surgeon to the Pennsylvania Hospital. He had received his appointment in 1794; consequently he served the institution twenty-two years. Some time previous to this, he had resigned his situations in the Philadelphia Dispensary, and in the Alms House Infirmary.

In the year 1819, Dr. Physick resigned his chair of Surgery in the University of Pennsylvania, and was transferred to that of Anatomy; which had become vacant the preceding session, by the death of his nephew, Dr. John Syng Dorsey.

The premature death of the lamented Dorsey plunged Dr. Physick into the deepest affliction, and had the effect of creating a melancholy gloom, which overshadowed the remainder of his existence. Dorsey, of all others, was most pre-eminently fitted to cheer and solace the declining years of his uncle. He had been regularly educated under the immediate inspection and superintendence of Dr. Physick; had imbibed from him his early lessons of wisdom and of knowledge, and at a more matured period of his life, adopted to the fullest extent his principles and doctrines. Advantages like these, aided by talents of a brilliant and comprehensive order, enabled Dorsey, at an unusually early period of his life, to assume the most elevated and distinguished rank in his profession. Relentless death, however, seized upon his prey, whilst in the midst of his honours and his usefulness.

It was always a source of deep regret with Dr. Physick's immediate family and friends, that his comforts in the evening of his days, and whilst labouring under physical infirmities, should be so greatly interrupted by translating him from the chair of Surgery to that of Anatomy. We had positive assurances from himself, that the change was contrary to his own wishes and inclination: how far the interests of the institution, to which he belonged, may have been promoted by it, I do not mean to inquire. My own impression is, however, and I believe I am not singular in the opinion, that if he had continued in the chair of Surgery, up to the period when he retired from the University, it would have numbered in its catalogue of students many more than it has ever shown.

In the Philadelphia Journal of the Medical and Physical Sciences, edited by Professor Chapman, vol. i., for the year 1820, Dr. Physick published a communication, in which he gave an account of the method which he employed for the removal of scirrhus tonsils, and hæmorrhoidal tumours, by means of the double canula and a soft wire. His method consisted in strangulating the tumours completely, by means of the wire ligature passed through a double canula; after which, instead of allowing the instrument to remain applied, as was formerly the custom, until the parts separated and were thrown off, a process requiring a week or ten days, it was his practice to remove the wire at the expiration of twenty-four hours. Ample experience has shown that this manner of removing these parts must be considered a valuable improvement and modification of the old method of permitting the ligature to remain on the parts until they sloughed away. We can readily imagine that the long continued irritation kept up by the instrument, would be productive of a degree of pain and suffering from which it is desirable to free the patient as soon as possible.

Some few years subsequently to this communication, he became convinced that the best and most convenient method of removing scirrhous tonsils, consisted in their excision. He contrived a very ingenious instrument for this purpose, and also for excising the uvula; a full description of which, accompanied with a plate, was published by Dr. Hays, in the American Journal of the Medical Sciences, vol. i.; together with the very interesting case of a young lady, afflicted with a most obstinate cough; occasioned by an elongation of the uvula, who was entirely cured by Dr. Physick, by means of the excision of a portion of that organ. In vol. ii., of the same Journal, Dr. Hays, its editor, published the description and plate of a forceps, invented by Dr. Physick, and employed in certain cases to facilitate the extirpation of the tonsil, by means of his instrument. The forceps is so constructed, that "the tonsil may be seized and drawn through the aperture to any distance that may be deemed proper; when its extirpation can be immediately effected."

It is proper that I should state, that in cases of hæmorrhoidal tumour, where the complaint was of long standing, when the lining membrane of the rectum was much diseased, and where the tumours were seated internally, Dr. Physick employed the ligature for their removal, as long as he continued to operate. Under the circumstances just mentioned, he considered this method of operating far safer than using the knife, and greatly to be preferred.

The following extract, taken from his communication on the use of the double canula and a wire, conveys a correct idea of his views upon this subject. "I have for many years been in the habit of performing the same kind of operation for the extirpation of hæmorrhoidal tumours. The canula used in this case should not be longer than about two inches. When hæmorrhoidal tumours are external and troublesome to the patient, almost all surgeons, I believe, cut them off; but when their attachments are within the anus, and the tumour only protrudes in the act of evacuating the fæces, then their excision would be attended with great risk of hæmorrhage. This some have denied, but having twice witnessed the fact to a very alarming extent, I wish, on all such occasions, to guard against it. The extirpation of such tumours can be performed safely by means of a ligature, of either vegetable or animal substance; but the most convenient and effectual I have ever tried, is a wire drawn at once tight round its base, by means of the double canula. This gives momentary pain, but it is not in all cases so severe as might be supposed. I am not able to account for this circumstance; but some patients make no complaint whatever, even though two or three tumours are operated on at the same time, while others exclaim violently from its intensity. At the end of twenty-four hours, and probably sooner, the wire may be removed in the manner above explained. The tumour will be found shrivelled and black, and in a few days will be separated and thrown off, under the application of a soft poultice of bread and milk."

Much has been said respecting the intensity of the pain accompanying the application of a ligature to hæmorrhoidal tumours. I have, however, repeatedly performed this operation, and not unfrequently the patients have expressed surprise at the little suffering which they experienced. Dr. Physick frequently related to me the case of a gentleman on whom he performed two operations for the removal of hæmorrhoidal tumours. In the one he used the knife, and in the other the ligature; and the patient declared that the knife caused him much greater pain than the application of the ligature. It is proper to mention, however, that in order to lessen the amount of pain, Dr. Physick considered it extremely important to include within the ligature nothing but the hæmorrhoidal tumour itself. It is undeniable that, in certain cases, the excision of hæmorrhoidal tumours is attended with the risk of fatal hæmorrhage. It is well known that cases have been reported by the highest authority in surgery, in which this operation has been attended with the loss of life.

I should suppose that Baron Dupuytren's cautions respecting this operation, in conjunction with his directions for the suppression of the hæmorrhage attendant upon it, would be quite sufficient to deter a majority of surgeons from excising internal hæmorrhoids.

The last paper written by Dr. Physick, which I shall briefly notice, is one which he published in vol. iii. of the Philadelphia Journal of the Medical and Physical Sciences, in which he communicated the particulars of a case of carbuncle, with some remarks on the use of the common caustic vegetable alkali in the treatment of this disease. In order for the better comprehension of his views respecting the use of the caustic, he divides the progress of carbuncle into three stages. The first or forming stage is that in which the peculiar inflammation exists in the cellular texture under the skin. The second stage is that in which the inflammation has terminated in the mortification of the parts. In the third stage an ulcer remains, attended, however, with no peculiarities.

He says, "In the first stage, all irritating treatment appears to be injurious, by increasing the peculiar inflammation then existing, and thereby extending it."

"In the second stage, the inflammation having ended in the death of the cellular texture in which it was situated, a process begins for making an opening through the skin, to allow the dead parts and acrid fluids to pass out. The commencement of this process is pointed out by the appearance of pimples and small orifices, as above described; and it is at this period that the application of caustic vegetable alkali upon the skin so perforated, and on that covering the middle of the tumour, in quantity sufficient to destroy it completely, proves highly beneficial. In all the cases in which I have used the caustic in this manner, the suffering of the patient ceased, as in Mr. Wharton's case, as soon as the pain from the caustic subsided. It operates by destroying in a few minutes that portion of the skin covering the mortified parts, which, if left to be removed by ulceration, would require several days for its completion, occasioning the chief part of the pain and danger attendant on and consequent to the disease."

In the year 1821, Dr. Physick was appointed Consulting Surgeon to the Institution for the Blind.

In 1822, the Phrenological Society of Philadelphia elected him its President.

In 1824, he was chosen President of the Philadelphia Medical Society. He held this situation until the time of his death.

In 1825, January 6, he was appointed a Member of the Royal Academy of Medicine of France; being, as far as I know, the first American who ever received that honour.

In 1831, in consequence of his declining health, he felt it incumbent on him to retire from the active duties of the University; and accordingly he resigned his situation as Professor of Anatomy. In acknowledgment of the extraordinary services which he had rendered, in elevating the character of the school, and in promoting the advancement of medical science, the institution, upon accepting his resignation, conferred upon him the highest honour in its power, by electing him unanimously "Emeritus Professor of Surgery and Anatomy."

Not the least among the improvements effected by Dr. Physick in the methods of treating diseases, may be considered his management of affections of the joints; and more especially that condition of the hip joint, known by the name of "morbus coxarius, or hip disease."

I may mention generally, that his practice consisted in the application of a carved splint, which would keep the limb strictly at rest, and prevent the least possible motion of the joint; and also in the prosecution of a course of active and long continued purging.

In the American Journal of the Medical Sciences, No. xiv., February, 1831, I published a detailed account of Dr. Physick's method of treating morbus coxarius, accompanied with a plate, exhibiting the application of the carved splint. The superiority of this method of treatment is now so completely established in this country as to lead to its adoption by the profession generally.

In October, 1831, Dr. Physick performed the operation of lithotomy on Chief Justice Marshall. This case was attended with singular interest, in consequence of the exalted position of the patient, his advanced age, and the circumstance of there being upward of one thousand calculi taken from his bladder. It is well known that for several years previous to this period, Dr. Physick had declined performing extensive surgical operations. He felt somewhat reluctant to operate upon Chief Justice Marshall, and offered to place the case in my hands. Taking all the circumstances into consideration, and knowing well that this would be the last time that he would ever perform a similar operation, I felt desirous that he should finish with so distinguished an individual; and accordingly urged him to do it himself. Upon the day appointed, the Doctor performed the operation with his usual skill and dexterity. I do not think I ever saw him display greater neatness than on that occasion. The result of the operation was complete success.

It will be readily admitted that, in consequence of Judge Marshall's very advanced age, the hazard attending the operation, however skilfully performed, was considerably increased. I consider it but an act of justice, due to the memory of that great and good man, to state, that in my opinion, his recovery was in a great degree owing to his extraordinary self-possession, and to the calm and philosophical views which he took of his case, and the various circumstances attending it.

It fell to my lot to make the necessary preparations. In the discharge of this duty I visited him on the morning of the day fixed on for the operation, two hours previously to that at which it was to be performed. Upon entering his room, I found him engaged in eating his breakfast. He received me with a pleasant smile upon his countenance, and said, "Well, Doctor, you find me taking breakfast, and I assure you I have had a good one. I thought it very probable that this might be my last chance, and therefore I was determined to enjoy it and eat heartily." I expressed the great pleasure which I felt at seeing him so cheerful, and said that I hoped all would soon be happily over. He replied to this, that he did not feel the least anxiety or uneasiness respecting the operation or its results. He said that he had not the slightest desire to live, labouring under the sufferings to which he was then subjected; that he was perfectly ready to take all the chances of an operation, and he knew there were many against him; and that if he could be relieved by it he was willing to live out his appointed time, but if not, would rather die than hold existence accompanied with the pain and misery which he then endured.

After he had finished his breakfast, I administered to him some medicine: he then inquired at what hour the operation would be performed. I mentioned the hour of eleven. He said, "Very well; do you wish me now for any other purpose, or may I lie down and go to sleep?" I was a good deal surprised at this question, but told him that if he could sleep it would be very desirable. He immediately placed himself upon the bed and fell into a profound sleep, and continued so until I was obliged to rouse him in order to undergo the operation.

He exhibited the same fortitude, scarcely uttering a murmur, throughout the whole procedure, which, from the peculiar nature of his complaint, was necessarily tedious.

Chief Justice Marshall survived this operation some years, and finally died of a disease of an entirely different character. Previously to his death he laboured under very unpleasant symptoms, which are frequently met with in advanced life; and in consequence of these, a rumour was widely disseminated that he had a recurrence of his old complaint, stone in the bladder. As this was the last operation of much magnitude performed by Dr. Physick, I feel desirous that it should be correctly estimated; and, inasmuch as I am still not unfrequently asked whether Judge Marshall had not a return of the calculus, I insert the following letter, addressed by Professor Chapman and myself to the editor of the Southern Literary Messenger, in order to correct an erroneous impression of this nature, given by an article in a previous number of that able Journal.

*Philadelphia, March* 25, 1836.

Sir:—A mistake, evidently unintentional, having appeared in the February number of your Journal for this year, we feel convinced you will, upon proper representation, take pleasure in correcting it; as an impression so erroneous might have a prejudicial tendency. Under the notice of the Eulogies on the Life and character of the late Chief Justice Marshall, it is there stated that, "for several years past Judge Marshall had suffered under a most excruciating malady. A surgical operation, by Dr. Physick, of Philadelphia, at length procured him relief; but a hurt received in travelling last spring seems to have caused a return of the former complaint with circumstances of aggravated pain and danger. Having revisited Philadelphia in the hope of again finding a cure, his disease there overpowered him; and he died on the 6th of July, 1835, in the 80th year of his age."

Now, sir, the above quotation is incorrect in the following respect. Judge Marshall never had a return of the complaint for which he was operated upon by Dr. Physick. After the demise of Chief Justice Marshall, it became our melancholy duty to make a post mortem examination, which we did in the most careful manner, and ascertained that his bladder did not contain one particle of calculous matter. Its mucous coat was in a perfectly natural state, and exhibited not the slightest traces of irritation.

The cause of his death was a very diseased condition of the liver, which was enormously enlarged, and contained several tuberculous abscesses of great size. Its pressure upon the stomach had the effect of dislodging this organ from its natural situation, and compressing it in such a manner, that for some time previous to his death it would not retain the smallest quantity of nutriment. By publishing this statement, you will oblige

Yours, very respectfully,
N. Chapman, M. D.
J. Randolph, M. D.

To J. W. White, Esq.

I should state, that at an early period after Judge Marshall's case, the operation of lithotripsy was introduced into this country. Dr. Physick became convinced of the extraordinary advantages which it possessed over that of lithotomy, and yielded it the full support of his sanction and approbation.

Among other contributions made by Dr. Physick to the department of surgery, I should mention that we are indebted to him for making us acquainted with the existence of preternatural pouches, or sacs, situated at the lower extremity of the rectum, just above the verge of the anus. This form of disease, which is one of not unfrequent occurrence, is in many instances productive of the most severe and distressing symptoms; so much so, that we have known patients labouring under it declare that their lives were scarcely supportable. The complaint is rendered more perplexing also from the almost uniform absence of all visible or external signs by which it may be designated. It is only by a peculiar mode of examination that its existence can be detected.

Those who wish to acquaint themselves more particularly with this disease, I refer to the "American Cyclopedia of Practical Medicine and Surgery," edited by Dr. Hays; in which is published, under the head of Anus, a most able article, written by my friend Dr. Reynell Coates, giving a minute and correct account of the nature and treatment of these preternatural pouches, as collected from Dr. Physick himself.

Before concluding the account of Dr. Physick's labours I may state, that in a conversation with his relative, Dr. R. R. Dorsey, a short time since, he recalled to my remembrance a case in which Dr. Physick had been eminently successful in alleviating, by means of a novel contrivance, the sufferings of a patient labouring under an enlargement of the prostate gland. As Dr. Dorsey attended this patient in conjunction with Dr. Physick, and had a particular knowledge of his method of procedure, I requested him to furnish me with an account of the case. He kindly acceded to my wishes, and sent me the following letter.

*Dear Doctor*,—I furnish, as desired, a description of an instrument invented and used by Dr. Physick, in 1835, in the case of a gentleman aged seventy, who had suffered for years from an enlargement of the third lobe of the prostate gland.

Very truly yours,
R. R. Dorsey.

*January* 12, 1839.

"The end of a small flexible catheter was introduced nearly to the bottom of a very thin sac or pouch, three inches long, and an inch and a half in diameter at the mouth. The edges of the sac, which was prepared from the intestine of a sheep, were secured to the catheter by a fine silk thread, wrapped around it with great care; and the material being as fine as the thinnest blotting paper, adapted itself, when oiled, so closely to the instrument, that the bulk of the whole was less than that of a large sized bougie.

"After its introduction into the bladder, the membrane was injected with tepid water, and the mouth of the catheter being stopped with a peg, it was gently, but with some firmness, retracted. The consequent pressure at the seat of disease, gentle and uniform, and from the nature of the material used, as little irritating as possible, had the happiest effect in repressing the enlarged lobe of the gland; and afforded for many months, great relief by facilitating the discharge of the urine. Although the patient took a severe cold immediately after the operation, he did not suffer more than he had previously; and on recovering from its temporary influence, he experienced a relief long unknown. The introduction of the instrument was again practised after an interval of some months, with great advantage.

"Much nicety is requisite in securing the edges around the catheter, so that there may be no roughness to cause irritation during its retraction. It was also deemed proper to wind the end of the thread loosely round the catheter and secure it to the stopper. The material employed was prepared and may be procured in France."

Dr. Physick informed me that he had been equally successful in relieving another case by means of the same contrivance.

In November, 1836, he was elected an honorary fellow of the Royal Medical and Chirurgical Society of London. The conferring of this honour was a full acknowledgment of his exalted merits, and justly acquired reputation; and he did not affect to conceal the high gratification which he derived from its acceptance.

I have mentioned, in the former part of this memoir, that the first case recorded in his private journal, is one in which he performed the extraction of the crystalline lens. By a singular coincidence, it happened that the last operation ever performed by Dr. Physick was for cataract, and took place but a few months previously to his death. He, however, never saw his patient after completing the process; the attack which terminated his existence occurring on the afternoon of the same day.

I ought to mention, by way of apology for his engaging in any surgical operation whilst labouring under such feeble health, that the circumstances attending this case were exceedingly peculiar. The applicant was a foreigner; Dr. Physick had operated upon his eye a year previously, and the gentleman had remained in this city during a whole year for the purpose of having the procedure repeated by him. He consequently felt it incumbent upon him not to disappoint his patient; and he was not the man to shrink from the performance of what he believed to be his duty, notwithstanding, as he informed me, he was well aware that death was impatiently waiting for his victim.

The date at which he performed this operation was the 13th of August, 1837. I was present on the occasion, and watched him with the most intense anxiety. He was quite collected and firm, and his hand was steady; notwithstanding at the time he was labouring under great mental and physical suffering. Whilst witnessing this last expiring effort in the cause of afflicted humanity, I felt a melancholy conviction that this would be the final act of his professional life, and that I should never again behold him engaged in a surgical operation.

From this period his complaint went on increasing in intensity and violence. The symptoms of hydrothorax became developed to a most painful extent, and he suffered extreme agony from oppression at his chest and difficulty of breathing; so much so, that sometimes he became unable to lie down in his bed for whole nights together, but was obliged to stand upon the floor, supported by assistants. In consequence of his increasing illness, his old and well-tried friend and associate, Professor Chapman, was requested to visit him in consultation with myself. His malady, however, had assumed too uncontrollable a form; and it resisted the most strenuous efforts that professional skill and affectionate attention could exert in his behalf.

Some time previously to his death, anasarca took place; and in consequence of his remaining so much in the erect position, his lower extremities became enormously swollen and distended with serum. The integuments at length gave way, openings were formed, and these finally ulcerated and became gangrenous.

The Father of American Surgery expired without a struggle, on the morning of the 15th of December, 1837, at twenty minutes past 8 o'clock.

> "He gave his honours to the world again,
> His blessed part to heaven, and slept in peace."

—

To the preceding account of the professional labours of Dr. Physick, I have but little to add respecting his private life and character. It is in fact rendered less necessary for me to dwell upon this point in his history, inasmuch as in the several obituary notices of him which have appeared from different sources, ample justice has been accorded to him both as a man and a citizen. It is with feelings of the most sincere gratification that I proceed to mention the following eulogies which were pronounced subsequently to the demise of Dr. Physick; all of them expressive of the deep sense which was entertained of his profound acquirements and personal qualifications.

"A comprehensive minute, commemorative of Philip Syng Physick, M. D., Emeritus Professor of Anatomy and Surgery in the University of Pennsylvania," was prepared, under the instructiôns of the Board of Trustees of the University, by Wm. Meredith, Esq. This is replete with sentiments which fully comply with the resolution of the Board, "That a committee be appointed to prepare and present, at the next meeting of this Board, a comprehensive minute; to state the long connection of the deceased with this University, and to express the respect entertained for his able and faithful services as a teacher, for his eminence as a practitioner of medicine, and for the virtues which adorned his private character."

When the intelligence of Dr. Physick's death was received at Louisville, "resolutions were adopted by the faculty and class of the Louisville Medical Institute, to commemorate, by a discourse prepared for the purpose, the invaluable services and character of the deceased." The duty of preparing this discourse devolved upon Professor Charles Caldwell, one of the early friends and associates of Dr. Physick. He discharged the obligations imposed upon him with his usual skill and ability; and delivered a discourse highly gratifying to the friends and connections of Dr. Physick.

At the request of the American Philosophical Society, a Necrological Notice of Dr. Physick was prepared, and presented at a meeting held in May, 1838, by Professor Wm. E. Horner. From Prosessor Horner's long association with Dr. Physick in the chair of Anatomy, it will be conceded that he possessed peculiar advantages for the successful accomplishment of his task. It is well known, too, that he entertained an ardent affection for Dr. Physick; and he has accordingly borne ample testimony to his talents and acquirements.

We are also indebted to Professor Granville S. Pattison, of Jefferson Medical College, for a highly laudatory notice of Dr. Physick, contained in an introductory lecture delivered before his class, on the commencement of the session of 1838–9.

It must be admitted that, by the community at large, Dr. Physick's private character was but imperfectly understood. This was owing to the habit of perfect seclusion which he contracted, and to the slight intercourse, other than professional, which he permitted himself to enjoy with his fellow-citizens. It must not be supposed, however, that this isolation arose from moroseness of character or want of inclination to mingle with society. A satisfactory explanation may be afforded by the entire self-abandonment with which he devoted himself to his professional engagements. In my opinion, this formed one of the most striking and remarkable points in Dr. Physick's character. I doubt very much whether history could show an example of a more pure and absolute devotion to professional pursuits than he exhibited.

In consequence of the reasons just mentioned, he was supposed by some to be stern and unfeeling, and wanting in the kinder sympathies of our nature. There could not be a greater misapprehension. His feelings were tender and susceptible in the extreme; and could those persons who entertained an opposite opinion have been admitted behind the scenes, and to closer and more intimate relations with him, they would have acknowledged the great injustice they had done him in such a surmise. Many instances may be cited, were it expedient to occupy the necessary time, in order to illustrate Dr. Physick's extreme tenderness of feeling. At an early stage of his professional career, he performed a few experiments upon living animals, with the view of determining some physiological points. This formed a lasting subject of regret to him as long as he lived; and he could not divest his mind of the idea that he had been guilty of a useless as well as a wicked act of cruelty.

Previously to his performing important surgical operations, his feelings were so harrowed up, and he experienced so much anxiety, that it was the custom of his family to endeavour to prevail upon him to execute such operations as speedily as possible, in order to relieve his mind.

To those who only saw Dr. Physick as the bold and unflinching operator in surgery, his character might have appeared cold and unfeeling, and they might have thought him,

——"Unlike to other men,
A snow-crown'd peak of science, towering high;"

but to the few who knew him in his private circle the veil was withdrawn. It was in the gentle charities of domestic life, as the tender and affectionate parent, or the sympathising friend, that his true character became revealed, and his heart was felt to be keenly alive to the kindest and softest emotions of which human nature is susceptible. He never appeared so happy as when surrounded by his children and his family; and indeed I feel assured that this formed one of the greatest consolations to him in the midst of his protracted sufferings.

In his intercourse with his professional brethren Dr. Physick's conduct was regulated by the strictest principles of honour and integrity. Whenever he was called in consultation with other physicians, without inquiring how exalted or humble their positions might be, he was scrupulously careful to avoid saying or doing any thing which could wound their feelings, or prejudice them in the least in the estimation of their patients. He invariably stated his own opinions in a frank and manly manner, and was ever willing to pay due deference to the opinions of others. Upon all occasions he was happy and ready to confer upon his fellow practitioners the benefit of his advice and experience, whether the information desired had special relation to themselves, or to those under their charge. He was far removed above the meanness of interfering with the patients of others; and whenever he had it in his power to render a service to a younger member of the profession, by a word of encouragement or commendation, it was cheerfully bestowed.

It was impossible that a man possessed of a mind of so reflective and contemplative a character as his, should not turn with anxious solicitude to the doctrines of religion, and the contemplation of a future state. Religion constituted, in fact, the most engrossing subject which occupied his attention during the latter years of his life. How far he derived comfort and consolation from his religious studies, it is not for me to say. I am very certain, however, that a more pure and ardent seeker after divine truth I never knew. As an observer of the principles of strict integrity and morality, I believe it will be conceded that he was exemplary to a remarkable degree. He, however, arrogated nothing to himself from this source. He expressed to me, but a short period previous to his death, that he possessed no merits of his own to give him a claim to salvation. His humility and self-abasement upon the subject of religion were extreme; and he was always willing and ready to apply to any source, however humble it might be, provided he thought he could be enlightened and instructed by it.

His course of reading upon theology was very extensive; and unfortunately for him, he read many works of a conflicting and contradictory nature. The effect of this upon one who had, during all his life, been in search of indisputable evidences, was to create, at times, gloomy and desponding views. Yet for very many years of his life, he was in the uniform habit of perusing, every morning, a portion of the New Testament; and when, in consequence of his illness and increasing infirmities, he was incapable of so doing, his children were constantly employed in reading this and other works of devotion to him. During his last illness he derived great pleasure and satisfaction from the visits of his friend and pastor, Dr. Delancey; whose kind attentions toward him were unremitting. I feel assured that the hopes and promises of the Christian religion were the greatest sources of consolation to him in the closing hours of his life, and smoothed his passage to the tomb.

